# Smad4 SUMOylation is essential for memory formation through upregulation of the skeletal myopathy gene *TPM2*

**DOI:** 10.1186/s12915-017-0452-9

**Published:** 2017-11-28

**Authors:** Wei L. Hsu, Yun L. Ma, Yen C. Liu, Eminy H. Y. Lee

**Affiliations:** 1 Institute of Biomedical Sciences, Academia Sinica, Taipei 115 Taiwan; 2 Graduate Institute of Life Sciences, National Defense Medical Center, Taipei 114, Taiwan

**Keywords:** Smad4, SUMOylation, PIAS1, *TPM2* gene, Spatial learning and memory, Skeletal myopathy

## Abstract

**Background:**

Smad4 is a critical effector of TGF-β signaling that regulates a variety of cellular functions. However, its role in the brain has rarely been studied. Here, we examined the molecular mechanisms underlying the post-translational regulation of Smad4 function by SUMOylation, and its role in spatial memory formation.

**Results:**

In the hippocampus, Smad4 is SUMOylated by the E3 ligase PIAS1 at Lys-113 and Lys-159. Both spatial training and NMDA injection enhanced Smad4 SUMOylation. Inhibition of Smad4 SUMOylation impaired spatial learning and memory in rats by downregulating TPM2, a gene associated with skeletal myopathies. Similarly, knockdown of TPM2 expression impaired spatial learning and memory, while TPM2 mRNA and protein expression increased after spatial training. Among the TPM2 mutations associated with skeletal myopathies, the TPM2E122K mutation was found to reduce TPM2 expression and impair spatial learning and memory in rats.

**Conclusions:**

We have identified a novel role of Smad4 SUMOylation and TPM2 in learning and memory formation. These results suggest that patients with skeletal myopathies who carry the TPM2E122K mutation may also have deficits in learning and memory functions.

**Electronic supplementary material:**

The online version of this article (doi:10.1186/s12915-017-0452-9) contains supplementary material, which is available to authorized users.

## Background

Transforming growth factor-β (TGF-β) signaling is known to regulate various cellular functions, including cell proliferation, differentiation, and apoptosis, as well as immune responses [1–4]. The biological effects of TGF-β are likely associated with TGF-β receptor phosphorylation and TGF-β receptor phosphorylation-mediated Smad2 and Smad3 phosphorylation. Phosphorylated Smad2 and Smad3 undergo conformational changes, allowing Smad2/Smad3 heterodimers to complex with Smad4, with the resulting Smad complexes translocated to the nucleus [5–8]. These Smad complexes either bind DNA or function as transcriptional co-activators or co-repressors of other transcription factors, thereby regulating the expression of many genes downstream of TGF-β signaling [9–12].

Post-translational modification of proteins with a small ubiquitin-like modifier (SUMO) is an important mechanism in the regulation of various cellular functions [13, 14]. We previously showed that protein SUMOylation is important for long-term memory formation [15, 16]. Protein SUMOylation also plays a role in protecting against amyloid-beta toxicity and H_2_O_2_-induced cell apoptosis [17, 18]. SUMOylation of Smad4 was found to be enhanced by Ubc9 and the protein inhibitor of the activated STAT (PIAS) family proteins [19]. In addition, Smad4 SUMOylation by PIAS1 and PIASxβ is enhanced by TGF-β-induced p38MAPK activation [20]. Smad4 can be SUMO-modified at both Lsy-113 and Lys-159 in HeLa cells, with SUMOylation of Smad4 promoting its nuclear accumulation and metabolic stability [21, 22]. Zinc was shown to increase the interaction between PIAS1 and the Smad2/Smad4 complex and to enhance p21^WAF1/Cip1^ expression, resulting in cancer cell apoptosis [23]. Few studies to date have assessed Smad protein SUMOylation, and the studies on this topic that have been conducted were all performed in vitro or in cell lines.

Less is known about the role of Smad proteins in the nervous system. One study found that Smad3 was essential to the survival of progenitor cells in dentate gyrus neurons of adult mice [24]. Another report showed that Smad1/Smad5/Smad8 signaling was necessary for the development of the nervous system [25]. To our knowledge, there have been no studies showing the role and function of Smad SUMOylation in the nervous system. The aim of the present study was to investigate the role and mechanism of Smad4 SUMOylation in the hippocampus in long-term memory formation. This study also sought to identify the downstream genes regulated by Smad4 SUMOylation and to determine the role of such SUMOylation in learning and memory functions. We found that the blockade of Smad4 SUMOylation impaired spatial learning and memory formation through downregulation of *TPM2* expression. Knockdown of TPM2 expression also impaired learning and memory performance. Several *TPM2* mutations were found to be associated with skeletal myopathies [26]. One of these mutations, *TPM2E122K*, reduced TPM2 expression and impaired spatial learning and memory performance. These findings suggest that memory function may be impaired in patients with certain types of skeletal myopathy.

## Results

### Identification of candidate SUMO sites on Smad4 in cells

**Fig. 1:**
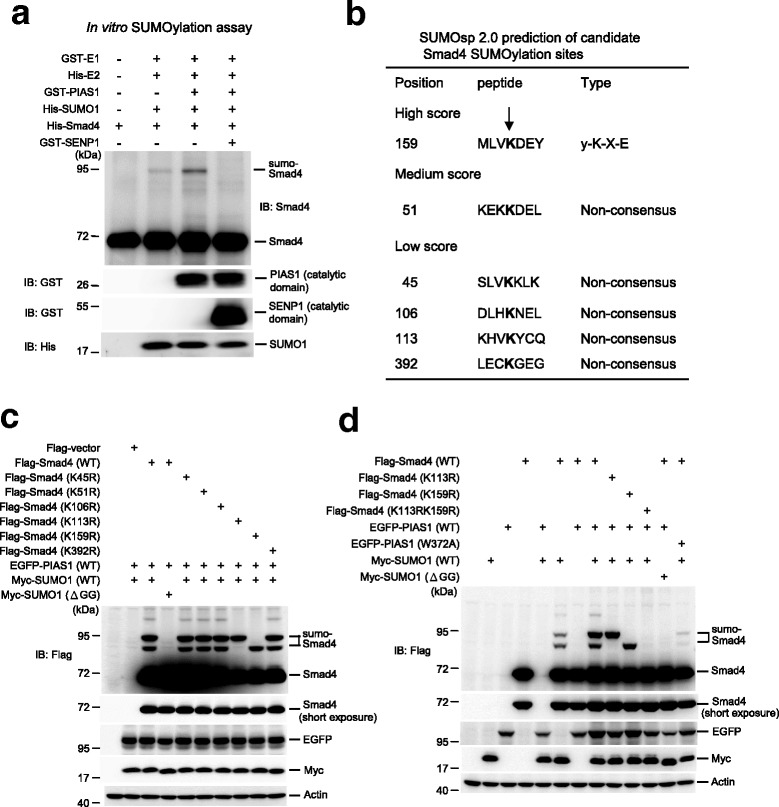
Identification of candidate SUMO sites on Smad4. **a** In vitro SUMOylation assay showing Smad4 SUMOylation by PIAS1. Purified GST-E1, His-E2, GST-PIAS1, His-SUMO1, His-Smad4, and GST-SENP1 proteins were added to the reaction for this assay. Various western blots were carried out. Experiments were performed in duplicate. **b** SUMO 2.0 Software prediction of candidate SUMO acceptors on Smad4. The letter K indicated by an arrow represents a candidate SUMO site. **c** EGFP-PIAS1 and Myc-SUMO1 (or Myc-SUMO1ΔGG) plasmids were co-transfected with Flag-vector, Flag-Smad4WT, or different Flag-Smad4 lysine mutant plasmids to HEK293T cells. Smad4 SUMOylation was examined 48 h later by immunoblotting with anti-Flag antibody. Western blots against enhanced green fluorescent protein (EGFP), Myc, and actin were also carried out. Experiments were performed in duplicate. **d** EGFP-PIAS1 (or EGFP-PIAS1W372A) and Myc-SUMO1 (or Myc-SUMO1ΔGG) plasmids were co-transfected with Flag-Smad4WT, Flag-Smad4K113R, Flag-Smad4K159R, or Flag-Smad4K113RK159R to HEK293T cells to confirm the candidate SUMO acceptors at Lys-113 and Lys-159. Smad4 SUMOylation was examined by immunoblotting using anti-Flag antibody 48 h later. Western blots against EGFP, Myc, and actin were also conducted. Experiments were performed in duplicate. EGFP enhanced green fluorescent protein

To determine whether Smad4 could be SUMO-modified by PIAS1, we performed in vitro SUMOylation assays. Recombinant E1, E2, and different His- or GST-tagged proteins were added to the SUMOylation reaction, followed by western blotting of the reaction products. Slight Smad4 SUMOylation was observed in the presence of E1, E2, SUMO1, and Smad4 proteins, but Smad4 SUMOylation was enhanced when the PIAS1 protein was also added. However, Smad4 SUMOylation was completely blocked by the addition of sentrin-specific protease 1 (SENP1), an enzyme that removes the SUMO molecule from SUMO-conjugated proteins (Fig. 1a). Further analysis using bioinformatics and SUMO 2.0 Software identified one lysine residue with a high score that also fits to the consensus SUMO-substrate motif ψ-K-X-E (Lys-159). Another lysine residue showed a medium score (Lys-51), and four showed low scores (Lys-45, Lys-106, Lys-113, and Lys-392), but none of these five residues showed a consensus SUMO-substrate motif (Fig. 1b). Based on these findings, we generated recombinant proteins with mutations in each of these six lysine residues and transfected each mutant plasmid (Flag-tagged), together with EGFP-PIAS1 and Myc-SUMO1 (or Myc-SUMO1ΔGG), into HEK293T cells. Smad4 SUMOylation was observed when plasmids bearing Flag-Smad4, EGFP-PIAS1, and Myc-SUMO1 were transfected (Fig. 1c, lane 3, upper and lower bands), but this effect was blocked by transfection of Myc-SUMO1ΔGG, a SUMO1 plasmid lacking the C-terminal di-glycine motif essential for SUMO1 conjugation [27] (Fig. 1c, lane 4). Transfection of Flag-Smad4K45R, Flag-Smad4K51R, Flag-Smad4K106R, and Flag-Smad4K392R did not alter Smad4 SUMOylation, whereas transfection of Flag-Smad4K113R blocked the lower Smad4 SUMO-band and transfection of Flag-Smad4K159R blocked the upper Smad4 SUMO-band (Fig. 1c).

Smad4 SUMOylation at K113 and K159 was confirmed by generating the Flag-Smad4K113RK159R double mutant and transfecting this plasmid into HEK293T cells, together with other plasmids, including EGFP-PIAS1W372A, a mutant that lacks PIAS1 E3 ligase activity [28]. Smad4 SUMOylation, which was observed when Flag-Smad4 and Myc-SUMO1 were transfected, was enhanced when EGFP-PIAS1 was also transfected (Fig. 1d). The lower and upper SUMO bands were blocked by transfection of Flag-Smad4K113R and Flag-Smad4K159R, respectively, whereas transfection of Flag-Smad4K113RK159R completely abolished Smad4 SUMOylation. Smad4 SUMOylation was also blocked when EGFP-PIAS1W372A was transfected (Fig. 1d). In addition, PIAS1 protein (indicated by the EGFP band) became more stable following PIAS1 SUMOylation of substrate protein (i.e. when EGFP-PIAS1 and Myc-SUMO1 were co-transfected), confirming our previous results [29].

### Smad4 is SUMO-modified by PIAS1 in the hippocampus

**Fig. 2:**
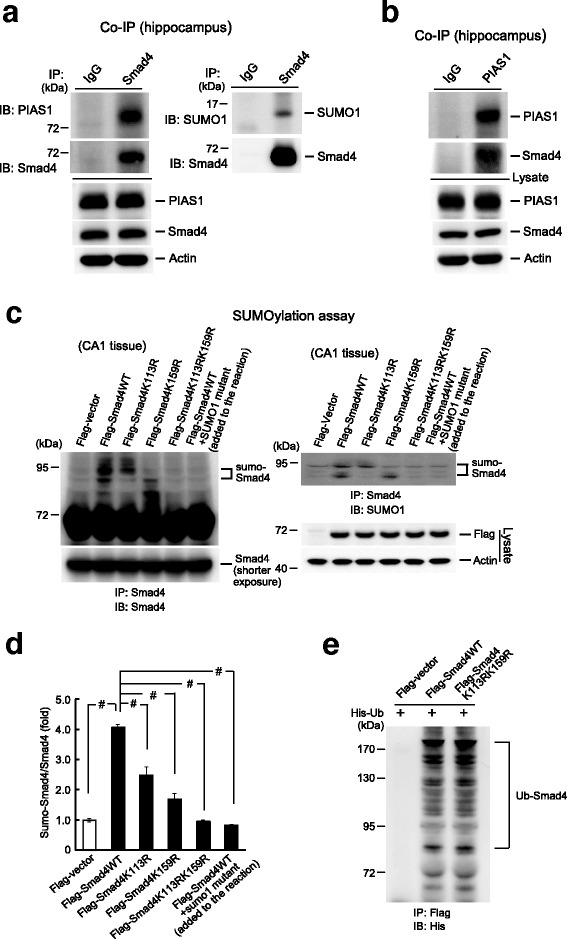
Smad4 is SUMO-modified by PIAS1 in the hippocampus. **a** Co-IP experiments showing that Smad4 is associated with PIAS1 (left panel) and SUMO1 (right panel) in the rat hippocampus. **b** Co-IP experiment showing that PIAS1 is associated with Smad4 in the rat hippocampus. **c** Flag-vector, Flag-Smad4WT (with or without the addition of SUMO1 mutant protein), and different Flag-Smad4 SUMO-mutant plasmids were transfected to the rat CA1 area and SUMOylation assay was carried out 48 h later to determine Smad4 SUMOylation at Lys-113 and Lys-159 in the hippocampus. Left panel: Immunoblotted with anti-Smad4 antibody. Right panel: The membrane was stripped and further immunoblotted with anti-SUMO1 antibody. Plasmid transfection and expression were confirmed by western blotting using anti-Flag antibody. **d** The quantified results. *n* = 5 for each group, *F*(5,24) = 65.09, ^#^ *P* < 0.001; *q* = 19.61, ^#^ *P* < 0.001 comparing the Flag-Smad4WT and Flag-vector groups; *q* = 10.1, ^#^ *P* < 0.001 comparing the Flag-Smad4WT and Flag-Smad4K113R groups; *q* = 15.27, ^#^ *P* < 0.001 comparing the Flag-Smad4WT and Flag-Smad4K159R groups; *q* = 19.92, ^#^ *P* < 0.001 comparing the Flag-Smad4WT and Flag-Smad4K113RK159R groups; and *q* = 20.82, ^#^ *P* < 0.001 comparing the Flag-Smad4WT and Flag-Smad4WT + SUMO1 mutant groups, using one-way ANOVA followed by post hoc Newman–Keuls multiple comparisons. Raw data and statistics are provided as Additional file 9. **e** Flag-vector, Flag-Smad4WT, or Flag-Smad4K113RK159R plasmid was co-transfected with His-ubiquitin plasmid to HEK293T cells. The anti-Flag antibody was used for immunoprecipitation and the anti-His antibody was used for immunoblotting. IB immunoblotting, IP immunoprecipitation, Ub-Smad4 ubiquitinated Smad4

In this series of experiments, we examined Smad4 SUMOylation by PIAS1 in the hippocampus. We first determined whether Smad4 is associated with PIAS1 and SUMO1 in the hippocampus by co-immunoprecipitation (co-IP). The use of anti-Smad4 antibody for immunoprecipitation and either anti-PIAS1 antibody or anti-SUMO1 for immunoblotting showed that Smad4 is associated with both PIAS1 (Fig. 2a, left panel) and SUMO1 (Fig. 2a, right panel). Similarly, the use of anti-PIAS1 antibody for immunoprecipitation and the anti-Smad4 antibody for immunoblotting also showed that PIAS1 is associated with Smad4 (Fig. 2b). These results suggest that Smad4 is probably SUMO-modified by PIAS1 in the brain. Next, we examined whether Smad4 could be SUMOylated at Lys-113 and Lys-159 by PIAS1 in the brain. Flag-vector, Flag-Smad4WT, Flag-Smad4K113R, Flag-Smad4K159R, or Flag-Smad4K113RK159R was transfected into the rat CA1 area and SUMOylation assays were performed 48 h later. Smad4 SUMOylation was observed when Flag-Smad4WT was transfected. Transfection of Flag-Smad4K113R abolished the lower SUMO-Smad4 band, transfection of Flag-Smad4K159R abolished the upper SUMO-Smad4 band, and transfection of the Flag-Smad4K113RK159R double mutant abolished both SUMO-Smad4 bands. Addition of the SUMO1-mutant protein also prevented Smad4 SUMOylation (Fig. 2c, left panel). These results were confirmed when the membrane was stripped and re-immunoblotted with anti-SUMO1 antibody (Fig. 2c, right panel). Plasmid transfection and expression were confirmed by western blotting with anti-Flag antibody (Fig. 2c, lower right panel). Quantification of Smad4 SUMOylation is shown in Fig. 2d.

We next assessed whether Smad4 is SUMOylated by endogenous PIAS1 in the brain. PIAS1 siRNA or control siRNA (8 pmol each) was transfected into the CA1 area and endogenous Smad4 SUMOylation (i.e., in the absence of the E1 and E2 enzymes, SUMO1, and recombinant PIAS1 protein) was determined 48 h later. Knockdown of PIAS1 significantly reduced the level of endogenous Smad4 SUMOylation (Additional file 1: Figure S1). Because SUMOylation and ubiquitination both occur at lysine residues, we further examined whether the observed SUMO-Smad4 bands could be ubiquitinated. Flag-vector, Flag-Smad4WT, or Flag-Smad4K113RK159R plasmid was co-transfected, along with His-ubiquitin plasmid, into HEK293T cells and the cell lysates were immunoprecipitated with anti-Flag antibody and immunoblotted with anti-His antibody. The ubiquitinated Smad4 bands were found to be similar in cells transfected with Smad4WT and Smad4K113RK159R (Fig. 2e).

### Spatial training and NMDA injection both increase Smad4 SUMOylation

**Fig. 3:**
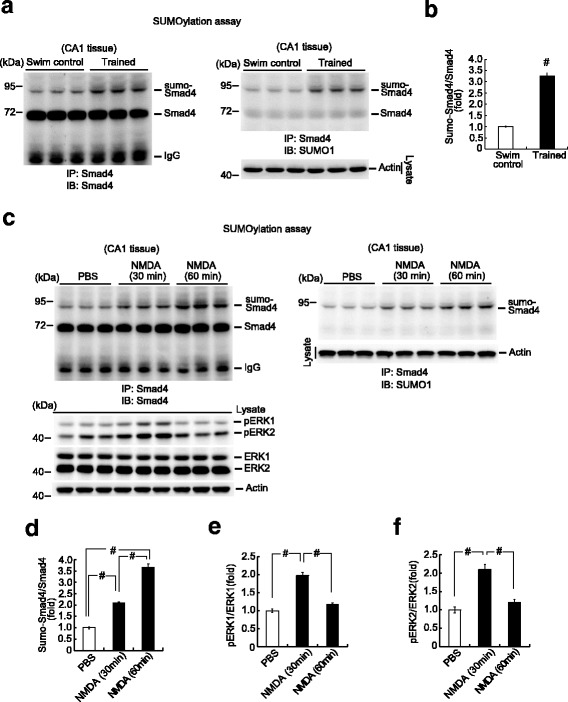
Spatial training and NMDA injection both increase Smad4 SUMOylation. **a** Rats either received water maze training for 4 days or served as swimming controls. They were sacrificed at the end of training and their CA1 tissue was subjected to Smad4 SUMOylation determination. Left panel: Immunoprecipitated with anti-Smad4 antibody and immunoblotted with anti-Smad4 antibody. Right panel: The membrane was stripped and further immunoblotted with anti-SUMO1 antibody. **b** Quantified results. *n* = 6 for each group, *t*(1,10) = 17.45, ^#^ *P* < 0.001, Student’s *t*-test. **c** Animals received an injection of PBS or NMDA (8 mM) to the CA1 area of their brains and were sacrificed 30 min or 60 min later. Their CA1 tissue was subjected to Smad4 SUMOylation assay and pERK1, pERK2, ERK1, and ERK2 determinations. Upper left panel: Immunoblotted with anti-Smad4 antibody. Right panel: The membrane was stripped and further immunoblotted with anti-SUMO1 antibody. Lower-left panel: Representative gel pattern of pERK1, pERK2, ERK1, and ERK2 western blot. **d** Quantified result of Smad4 SUMOylation. *n* = 6 for each group, *F*(2,15) = 220.72, ^#^ *P* < 0.001; *q* = 12.03, ^#^ *P* < 0.001 comparing the PBS and NMDA 30 min groups; *q* = 29.54, ^#^ *P* < 0.001 comparing the PBS and NMDA 60 min groups; *q* = 17.52, ^#^ *P* < 0.001 comparing the NMDA 30 min and NMDA 60 min groups. **e** Quantified result of pERK1/ERK1. *n* = 6 for each group, *F*(2,15) = 65.36, ^#^ *P* < 0.001; *q* = 15.14, ^#^ *P* < 0.001 comparing the PBS and NMDA 30 min groups; *q* = 12.48, ^#^ *P* < 0.001 comparing the NMDA 30 min and NMDA 60 min groups. **f** Quantified result of pERK2/ERK2. *n* = 6 for each group, *F*(2,15) = 27.71, ^#^ *P* < 0.001; *q* = 9.9, ^#^ *P* < 0.001 comparing the PBS and NMDA 30 min groups; *q* = 8.05, ^#^ *P* < 0.001 comparing the NMDA 30 min and NMDA 60 min groups. Data are expressed as means ± SEMs. IB immunoblotting, IP immunoprecipitation, PBS phosphate-buffered saline, SEM standard error of the mean

We next assessed whether Smad4 SUMOylation is associated with spatial learning. Rats were randomly divided into two groups, with one group subjected to water maze training and the other group serving as the swimming control. The rats were sacrificed at the end of training and their CA1 tissue was dissected out and subjected to SUMOylation assay (immunoblotted with anti-Smad4 antibody). Spatial training was found to increase the level of Smad4 SUMOylation (Fig. 3a, left panel). Stripping of the membrane and subsequent immunoblotting with anti-SUMO1 antibody yielded the same SUMO-Smad4 band (Fig. 3a, right panel). The quantification of Smad4 SUMOylation after training is shown in Fig. 3b.

The finding that spatial training increased Smad4 SUMOylation suggested that neuronal activation would also enhance Smad4 SUMOylation. The CA1 areas of rat brains were injected with phosphate-buffered saline (PBS) or 8 mM N-methyl-D-aspartate (NMDA). The animals were sacrificed 30 min or 60 min after injection and their CA1 tissue was dissected out and subjected to SUMOylation assays. Smad4 SUMOylation was increased 30 min after NMDA injection and further enhanced after 60 min (Fig. 3c, left panel). Similar results were observed when the membranes were stripped and re-immunoblotted with anti-SUMO1 antibody (Fig. 3c, right panel). The quantification of Smad4 SUMOylation after NMDA injection is shown in Fig. 3d. The neuronal activating activity of NMDA was confirmed by the increases in phospho-ERK1 and phospho-ERK2 expression 30 min after NMDA injection, with the levels of both returning to pretreatment conditions 60 min later. ERK1 and ERK2 levels remained unchanged (Fig. 3c, lower panel). The relationship between spatial training and Smad4 SUMOylation was also supported by the increased association between PIAS1 and Smad4 in trained animals compared with the swimming controls (Additional file 2: Figure S2).

Other than the important role that NMDA receptors play in neuronal plasticity, NMDA also induces excitotoxic damage in neurons. Therefore, we examined whether NMDA was excitotoxic to CA1 neurons in vivo using the TUNEL assay. Kainic acid treatment (0.4 μg) was used as a positive control. Immunohistochemical results revealed that injection of 0.7 μl of 8 mM NMDA (5.6 nmol) was not toxic to CA1 neurons after 30 min or 1 h (Additional file 3: Figure S3).

### Blockade of Smad4 SUMOylation impairs spatial memory formation

**Fig. 4:**
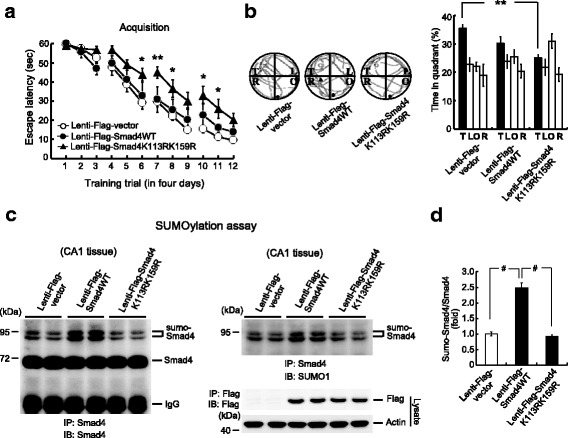
Blockade of Smad4 SUMOylation impairs spatial learning and memory formation. **a** Animals received lenti-Flag-vector, lenti-Flag-Smad4WT, or lenti-Flag-Smad4K113RK159R transduction and were subjected to water maze learning. *n* = 8 for each group, *F*(2,21) = 5.55, **P = 0.01; *q* = 4.14, *P < 0.05 comparing the lenti-Flag-Smad4K113RK159R and lenti-Flag-vector groups; *q* = 4.01, **P = 0.01 comparing the lenti-Flag-Smad4K113RK159R and lenti-Flag-Smad4WT groups. The statistical difference between the lenti-Flag-Smad4WT and lenti-Flag-Smad4K113RK159R groups for a given trial is indicated by the significance signs: *P < 0.05 and **P < 0.01. **b** Probe trial test. *n* = 8 each group, *F*(2,21) = 4.87, *P < 0.05; *q* = 4.41, **P = 0.01 comparing the lenti-Flag-Smad4K113RK159R and lenti-Flag-vector groups. **c** Animals were sacrificed after the probe trial test and their CA1 tissue was subjected to SUMOylation assay. Left panel: Immunoblotted with anti-Smad4 antibody. Right panel: Immunoblotted with anti-SUMO1 antibody. Lentivector transduction and expression was confirmed by western blotting using anti-Flag antibody. **d** Quantified result of Smad4 SUMOylation. *n* = 8 for each group, *F*(2,21) = 64.04, ^#^ *P* < 0.001; *q* = 13.51, ^#^ *P* < 0.001 comparing the lenti-Flag-vector and lenti-Flag-Smad4WT groups; *q* = 14.19, ^#^ *P* < 0.001 comparing the lenti-Flag-Smad4WT and lenti-Flag-Smad4K113RK159R groups. Data are expressed as means ± SEMs. IB immunoblotting, IP immunoprecipitation, SEM standard error of the mean

The increased Smad4 SUMOylation induced by spatial learning and neuronal activation suggested that a blockade of Smad4 SUMOylation should impair spatial learning and memory formation. To examine this hypothesis, rats were transduced with the lentivectors lenti-Flag-vector, lenti-Flag-Smad4WT vector, and lenti-Flag-Smad4K113RK159R vector and subjected to water maze learning 14 days later. Although transduction of lenti-Flag-Smad4WT had no effect on learning, transduction of lenti-Flag-Smad4K113RK159R markedly impaired learning (Fig. 4a). Similar results were obtained with the probe trial test, performed the next day. Animals transduced with lenti-Flag-Smad4K113RK159R spent less time in the target quadrant than those transduced with lenti-Flag-vector or lenti-Flag-Smad4WT (Fig. 4b, right panel). Representative swimming patterns of animals from each group are also shown (Fig. 4b, left panel). The rats were sacrificed after the probe trial test, and their CA1 tissue was dissected out and assayed for Smad4 SUMOylation. Overexpression of Smad4 was found to enhance Smad4 SUMOylation, an effect completely blocked by transduction of Smad4K113RK159R (Fig. 4c, left panel). The same result was observed when the membrane was stripped and re-immunoblotted with anti-SUMO1 antibody (Fig. 4c, right panel). Smad4 vector transduction and expression were confirmed by western blotting with anti-Flag antibody because all the lentivectors contained the Flag-tag (Fig. 4c, lower-right panel). Quantification of Smad4 SUMOylation is shown in Fig. 4d.

Smad4 is an important effector of TGF-β signaling [10]. In addition, Smad4 SUMOylation by PIAS1 was found to activate TGF-β signaling and enhance TGF-β-induced cellular responses [22, 30]. If the same mechanism occurs in the brain, then activation of TGF-β signaling in the hippocampus should facilitate spatial learning and memory, similar to Smad4 SUMOylation. This hypothesis was tested by injecting PBS or 1 μM SB525334, a TGF-β receptor inhibitor, into the CA1 areas of rat brains, followed by water maze learning and the probe trial test. Injection of SB525334 markedly impaired spatial acquisition and reduced the time animals spent in the target quadrant on the probe trial test (Additional file 4: Figure S4).

### Identification of TPM2 as a downstream target of Smad4 SUMOylation, with knockdown of TPM2 impairing spatial learning and memory

**Fig. 5:**
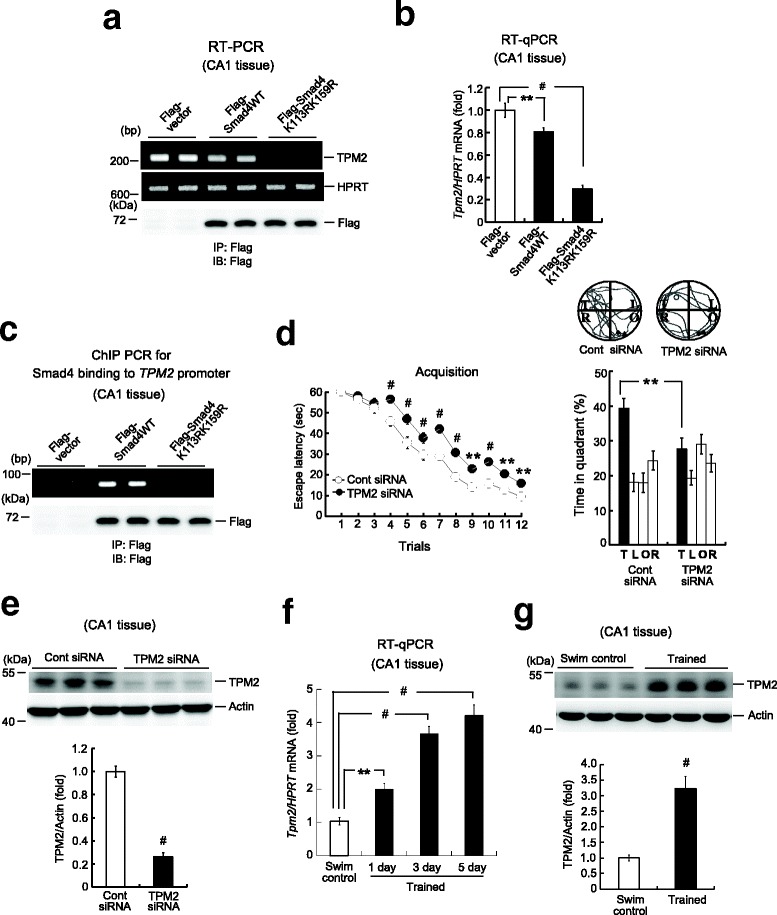
Identification of TPM2 as a downstream target of Smad4 SUMOylation. Knockdown of TPM2 impairs spatial learning and memory formation. **a** Animals received Flag-vector, Flag-Smad4WT, or Flag-Smad4K113RK159R transfection and their CA1 tissue was subjected to RT-PCR analysis of *TPM2* and *HPRT* gene expression 48 h later. Plasmid transfection and expression were confirmed by immunoprecipitation and immunoblotting with anti-Flag antibody. **b** Separate animals received the same plasmid transfections as described in (**a**) and their CA1 tissue was subjected to RT-qPCR analysis of *TPM2* mRNA expression. *n* = 7 for each group, *F*(2,18) = 337.56, ^#^ *P* < 0.001; *q* = 8.56, **P < 0.01 comparing the Flag-vector and Flag-Smad4WT groups; *q* = 35.23, ^#^ *P* < 0.001 comparing the Flag-vector and Flag-Smad4K113RK159R groups. **c** The same plasmids were transfected to the rat CA1 area, and direct Smad4 binding to the *TPM2* promoter was determined by ChIP PCR assay. Plasmid transfection and expression were confirmed by western blotting using the anti-Flag antibody. Experiments were performed in triplicate. **d** Animals received control siRNA or TPM2 siRNA transfection and were subjected to water maze learning and the probe trial test. *n* = 9 for each group, *F*(1,16) = 33.31, ^#^ *P* < 0.001; *q* = 5.77, ^#^ *P* < 0.001 between the control siRNA and TPM2 siRNA groups for spatial acquisition and *t*(1,16) = 2.98, **P < 0.01 for the probe trial test. A representative swim pattern from each group is also shown. The statistical difference between the control siRNA and TPM2 siRNA groups for a given trial is indicated by the significance signs: **P < 0.01 and ^#^ *P* < 0.001. **e** Animals (*n* = 9 for each group) were sacrificed after the probe trial test and their CA1 tissue was subjected to western blot analysis of TPM2 expression. The quantified results are also shown. *t*(1,16) = 23.69, ^#^ *P* < 0.001. **f** Different animals were subjected to water maze training for 1, 3, or 5 days. Another group served as swimming controls. They were sacrificed at the end of training and their CA1 tissue was subjected to RT-qPCR analysis of *TPM2* mRNA expression. *n* = 6 for each group, *F*(3,20) = 46.2, ^#^ *P* < 0.001; *q* = 4.43, **P < 0.01 comparing the control and 1-day training groups; *q* = 12.14, ^#^ *P* < 0.001 comparing the control and 3-day training groups; *q* = 14.69, ^#^ *P* < 0.001 comparing the control and 5-day training groups. **g** Animals were subjected to water maze training for 5 days or served as swimming controls. They were sacrificed at the end of training and their CA1 tissue was subjected to western blot analysis of TPM2 expression. The quantified result is also shown. *n* = 5 each group, *t*(1,8) = 5.52, ^#^ *P* < 0.001. Data are expressed as means ± SEMs. Raw data and statistics are provided as Additional file 9. ChIP chromatin immunoprecipitation, Cont control, IB immunoblotting, IP immunoprecipitation, RT-PCR reverse-transcription polymerase chain reaction, RT-qPCR reverse-transcription quantitative real-time polymerase chain reaction, SEM standard error of the mean

The downstream genes associated with spatial learning that are regulated by the SUMOylation of the transcription factor Smad4 were assessed by cDNA microarray analysis. Transfection of Flag-Smad4K113RK159R altered the expression of approximately 40,000 genes compared with the Flag-vector control. Among these genes, 1,199 showed more than twofold upregulation and 1,961 showed more than twofold downregulation. The ontological analysis of these genes is shown in Additional file 5: Table S1. Smad4K113RK159R transfection downregulated the expression of the *TPM2* gene 8.13-fold, a result confirmed by reverse-transcription polymerase chain reaction (RT-PCR) and reverse-transcription quantitative real-time polymerase chain reaction (RT-qPCR) analyses. RT-PCR showed that transfection of the Flag-Smad4WT plasmid slightly decreased the *TPM2* mRNA level, whereas transfection of the Flag-Smad4K113RK159R plasmid markedly decreased the *TPM2* mRNA level in the hippocampus (Fig. 5a). Plasmid transfection and expression were confirmed by western blotting with anti-Flag antibody (Fig. 5a, lower panel). RT-qPCR showed that transfection of Flag-Smad4WT reduced the *TPM2* mRNA level by approximately 20%, whereas transfection of Flag-Smad4K113RK159R plasmid markedly reduced the *TPM2* mRNA level in the hippocampus (Fig. 5b). Moreover, transfection of these Smad4 plasmids was found to have no effect on *HPRT* mRNA level, suggesting that the latter is an appropriate internal control (Additional file 6: Table S2).

We also assessed whether a blockade of Smad4 SUMOylation could prevent Smad4 from binding to the *TPM2* promoter in the brain. Flag-vector, Flag-Smad4WT, and Flag-Smad4K113RK159R plasmids were transfected into the rat CA1 area. The animals were sacrificed 48 h later and their CA1 tissue was subjected to chromatin immunoprecipitation (ChIP) assays. Smad4 was found to bind directly to the endogenous *TPM2* promoter, but the blockade of Smad4 SUMOylation completely abolished this effect (Fig. 5c). Plasmid transfection and expression were confirmed by western blotting with the anti-Flag antibody (Fig. 5c, lower panel).

Because *TPM2* is a downstream gene regulated by Smad4 SUMOylation and the blockade of Smad4 SUMOylation impairs spatial learning and memory formation, a reduced level of TPM expression should similarly impair spatial learning and memory. Because knockout of the *TPM* genes *TPM1*, *TPM2*, and *TPM3* is embryonically lethal [31], the effect of reduced TPM expression was assessed by RNA interference. Animals were randomly transfected with TPM2 or control siRNA and subjected to water maze learning and retention measures 48 h later. Transfection of TPM2 siRNA impaired spatial acquisition (Fig. 5d, left panel) and reduced the time animals spent in the target quadrant compared with control animals (Fig. 5d, right panel). A representative swim pattern from each group is also shown (Fig. 5d, upper right panel). Animals were sacrificed after the probe trial test and their CA1 tissue was subjected to a western blot analysis of TPM2 expression. Transfection of TPM2 siRNA was found to markedly reduce TPM2 expression in the CA1 area (Fig. 5e).

We further examined the role of TPM2 in spatial learning and memory formation by subjecting separate groups of animals to 1, 3, or 5 days of spatial training. These rats were sacrificed at the end of training and their CA1 tissue was subjected to RT-qPCR determination of the *TPM2* mRNA level. Training markedly increased *TPM2* mRNA levels, with the increase correlating with the number of days of training (Fig. 5f). In contrast, *HPRT* mRNA levels were not affected by the number of days of training (Additional file 7: Table S3). These results, suggesting that *TPM2* mRNA expression is involved in the memory consolidation process, were supported by the finding that 5-day spatial training dramatically increased the level of TPM2 expression in the CA1 area (Fig. 5g).

### TPM2 siRNA transfection blocks the facilitating effect of Smad4 SUMOylation on spatial learning and memory

**Fig. 6:**
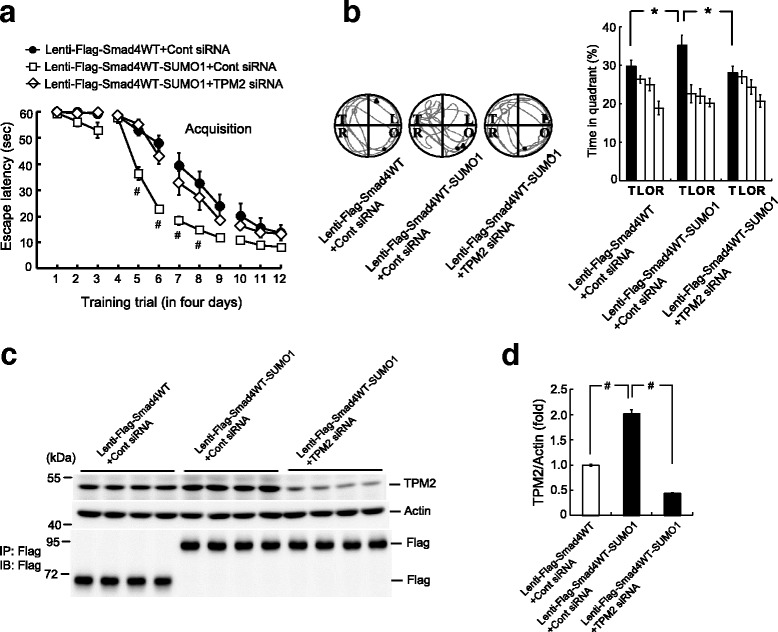
TPM2 siRNA transfection blocks the facilitating effect of Smad4 SUMOylation on spatial learning and memory. **a** Animals received lenti-Flag-Smad4WT + control siRNA, lenti-Flag-Smad4WT-SUMO1 + control siRNA, or lenti-Flag-Smad4WT-SUMO1 + TPM2 siRNA (4 pmol) transduction and were subjected to water maze learning. *n* = 8 for each group, *F*(2,21) = 11.94, ^#^ *P* < 0.001; *q* = 6.58, ^#^ *P* < 0.001 comparing the lenti-Flag-Smad4WT + control siRNA and lenti-Flag-Smad4WT-SUMO1 + control siRNA groups; *q* = 5.12, **P < 0.01 comparing the lenti-Flag-Smad4WT-SUMO1 + control siRNA and lenti-Flag-Smad4WT-SUMO1 + TPM2 siRNA groups. The statistical difference between the lenti-Flag-Smad4WT-SUMO1 + control siRNA and lenti-Flag-Smad4WT-SUMO1 + TPM2 siRNA groups for a given trial is indicated by the significance sign: ^#^ *P* < 0.001. **b** Probe trial test. *n* = 8 for each group, *F*(2,21) = 5.33, *P < 0.05; *q* = 3.48, *P < 0.05 comparing the lenti-Flag-Smad4WT + control siRNA and lenti-Flag-Smad4WT-SUMO1 + control siRNA groups; *q* = 4.37, *P < 0.05 comparing the lenti-Flag-Smad4WT-SUMO1 + control siRNA and lenti-Flag-Smad4WT-SUMO1 + TPM2 siRNA groups. **c** Animals were sacrificed after the probe trial test and their CA1 tissue was subjected to TPM2 determination by western blotting. Lentivector transduction and expression were confirmed by western blotting using the anti-Flag antibody. **d** Quantified results of TPM2 expression. *n* = 8 for each group, *F*(2,21) = 268.4, ^#^ *P* < 0.001; *q* = 20.76, ^#^ *P* < 0.001 comparing the lenti-Flag-Smad4WT + control siRNA and lenti-Flag-Smad4WT-SUMO1 + control siRNA groups; *q* = 32.33, ^#^ *P* < 0.001 comparing the lenti-Flag-Smad4WT-SUMO1 + control siRNA and lenti-Flag-Smad4WT-SUMO1 + TPM2 siRNA groups. Data are expressed as means ± SEMs. IB immunoblotting, IP immunoprecipitation, SEM standard error of the mean

Although our results revealed that the blockade of Smad4 SUMOylation and knockdown of TPM2 expression both impaired spatial learning and memory performance, these results did not show whether TPM2 mediates the effect of Smad4 SUMOylation on spatial learning and memory. To examine this, rats were transduced with mixtures of lenti-Flag-Smad4WT vector + control siRNA, lenti-Flag-Smad4WT-SUMO1 vector + control siRNA, or lenti-Flag-Smad4WT-SUMO1 vector + TPM2 siRNA (4 pmol) and subjected to water maze learning 14 days later and probe trial tests the next day. Rats injected with lenti-Flag-Smad4WT-SUMO1 vector + control siRNA showed improved spatial acquisition compared with rats injected with lenti-Flag-Smad4WT vector + control siRNA. However, the facilitating effect of Smad4WT-SUMO1 transduction on spatial acquisition was blocked by co-transfection of TPM2 siRNA (Fig. 6a). Similar results were obtained with the probe trial test. Animals injected with lenti-Flag-Smad4WT-SUMO1 vector + control siRNA spent more time in the target quadrant than animals injected with lenti-Flag-Smad4WT vector + control siRNA, an effect that was also blocked by co-transfection of TPM2 siRNA (Fig. 6b, right panel). Representative swim patterns of animals from each group are also shown (Fig. 6b, left panel).

The animals were sacrificed after the probe trial test and their CA1 tissue was dissected out for determination of TPM2 expression. Western blotting showed that the level of TPM2 expression was significantly higher in rats transduced with the Smad4WT-SUMO1 lentivector than with the Smad4WT lentivector, but that co-transfection of TPM2 siRNA completely blocked the effect of Smad4WT-SUMO1 transduction on TPM2 expression (Fig. 6c). Smad4 lentivector transduction and expression were confirmed by western blotting with anti-Flag antibody (Fig. 6c, lower panel). A quantification of TPM2 expression is shown in Fig. 6d.

### Spatial training selectively increases Smad3 phosphorylation and its association with Smad4

**Fig. 7:**
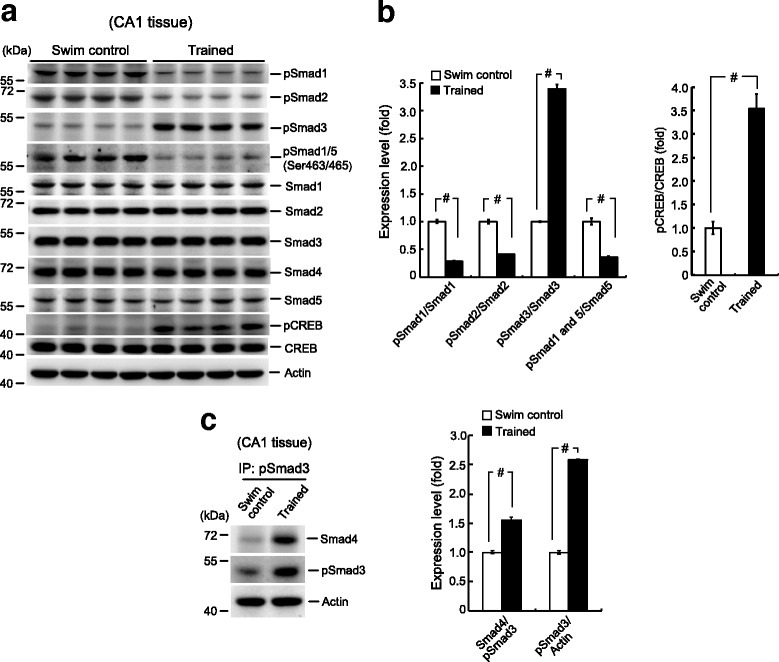
Spatial training selectively increases Smad3 phosphorylation and its association with Smad4. **a** Animals were subjected to spatial training for 1 day or served as swimming controls. They were sacrificed at the end of training and their CA1 tissue was subjected to western blot analysis of various Smad, pSmad, CREB, and pCREB expression levels. Representative gel patterns are shown. **b** Quantified results. *n* = 4 for each group, *t*(1,6) = 21.26, ^#^ *P* < 0.001 for pSmad1/Smad1; *t*(1,6) = 16.29, ^#^ *P* < 0.001 for pSmad2/Smad2; *t*(1,6) = 28.76, ^#^ *P* < 0.001 for pSmad3/Smad3; *t*(1,6) = 10.24, ^#^ *P* < 0.001 for pSmad5/Smad5; *t*(1,6) = 7.41, ^#^ *P* < 0.001 for pCREB/CREB. Raw data and statistics are provided as Additional file 9. **c** Co-IP experiment showing the relationship between pSmad3 and Smad4 in trained (1 day) and swimming control animals. Experiments are in four repeats and the quantified results are shown. *n* = 4 each group, *t*(1,6) = 8.96, ^#^ *P* < 0.001 for Smad4/pSmad3 and *t*(1,6) = 47.37, ^#^ *P* < 0.001 for pSmad3/actin. Raw data and statistics are provided as Additional file 9. Data are expressed as means ± SEMs. IP immunoprecipitation, SEM standard error of the mean

The above results demonstrate that Smad4 SUMOylation is necessary for spatial learning and memory formation. Next, we studied its underlying mechanism. Smad family proteins, except for Smad4, are phosphorylated upon stimulation. These phosphorylated proteins form homo- or hetero-dimers, which bind to the co-Smad protein Smad4. This Smad complex subsequently translocates to the nucleus, where it regulates transcription and is otherwise modified. To identify the Smad protein that associates with Smad4 prior to Smad4 SUMOylation during memory formation, we first attempted to identify the Smad protein activated during spatial learning. Animals were randomly divided into two groups, with one group receiving water maze training for 1 day and the other serving as swimming controls. The rats were sacrificed after training, and their CA1 tissue was dissected out and subjected to western blotting analysis of various phospho- (p)-Smad proteins and Smad proteins. The level of phospho-cyclic AMP-responsive element binding protein (CREB) was also determined to confirm the effectiveness of water maze training. We found that the levels of pSmad1, pSmad2, and pSmad1/5 were significantly reduced, while the level of pSmad3 was markedly increased after training. The expression levels of the non-phosphorylated Smad proteins remained unchanged (Fig. 7a). The quantified results are shown in Fig. 7b, left panel. Spatial training also increased the pCREB level, but CREB expression was unaltered (Fig. 7a and b, right panel).

These results suggested that training selectively increased Smad3 phosphorylation in the CA1 area. To determine whether training also increased the association between pSmad3 and Smad4 before Smad complex translocation and Smad4 SUMOylation, a co-IP experiment was performed. The level of Smad3 phosphorylation and the association between pSmad3 and Smad4 were found to be higher after training than in the swimming controls (Fig. 7c, left panel). The quantified results are shown in Fig. 7c, right panel.

### The *TPM2E122K* mutant decreases TPM2 expression and impairs spatial learning and memory formation

**Fig. 8:**
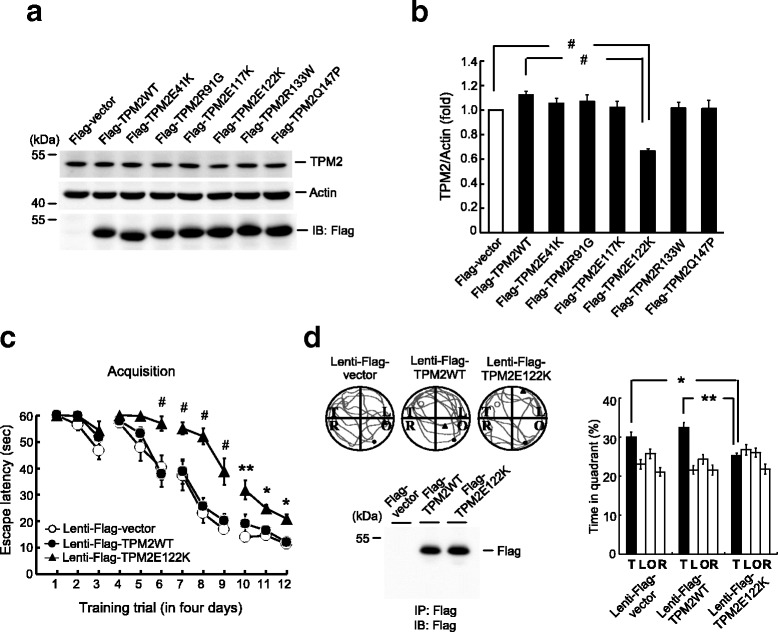
The *TPM2E122K* mutant decreases TPM2 expression and impairs spatial learning and memory formation. **a** Flag-vector, Flag-TPM2WT, and various Flag-TPM2 mutant plasmids were transfected to HEK293T cells and TPM2 expression was determined by western blotting. Western blotting with anti-Flag antibody was used to confirm similar amounts of transfection and expression for each plasmid. **b** Quantified result of TPM2 expression. *n* = 5 for each group, *F*(7,32) = 9.09, *P* > 0.05; *q* = 7.25, ^#^ *P* < 0.001 comparing the TPM2E122K and control groups; and *q* = 9.91, ^#^ *P* < 0.001 comparing the TPM2E122K and TPM2WT groups. Raw data and statistics are provided as Additional file 9. **c** Animals received lenti-Flag-vector, lenti-Flag-TPM2WT, or lenti-Flag-TPM2E122K mutant vector transduction to the CA1 area of their brains and were subjected to water maze learning. *n* = 8 for each group, *F*(2,21) = 19.17, ^#^ *P* < 0.001; *q* = 8.22, ^#^ *P* < 0.001 comparing the TPM2E122K and control groups; and *q* = 6.73, ^#^ *P* < 0.001 comparing the TPM2E122K and TPM2WT groups. The statistical difference between the lenti-Flag-TPM2WT and lenti-Flag-TPM2E122K groups for a given trial is indicated by the significance signs: *P < 0.05, **P < 0.01 and ^#^ *P* < 0.001. **d** Probe trial test. *n* = 8 for each group, *F*(2,21) = 11.09, ^#^ *P* < 0.001; *q* = 4.34, *P < 0.05 comparing the TPM2E122K and control groups; and *q* = 6.55, **P < 0.01 comparing the TPM2E122K and TPM2WT groups. A representative swim pattern from each group is also shown. Data are expressed as means ± SEMs. IB immunoblotting, IP immunoprecipitation, SEM standard error of the mean

The *TPM2* gene encodes the protein β-tropomyosin, which is mainly expressed in skeletal muscle. Although mutations in *TPM2* have been associated with different myopathies [26], it is not known whether these mutations alter the expression of TPM2. Plasmids encoding six commonly observed *TPM2* mutations, *TPM2E41K*, *TPM2R91G*, *TPM2E117K*, *TPM2E122K*, *TPM2R133W* and *TPM2Q147P* [26], were transfected into HEK293T cells, and TPM2 expression was examined by western blotting. The only mutant with an altered TPM2 expression level was *TPM2E122K*, which showed 40% lower TPM2 expression than *TPM2WT* (Fig. 8a and b). Similar levels of plasmid transfection and expression were confirmed by western blotting with anti-Flag antibody (Fig. 8a), with the quantified results shown in Fig. 8b.

To assess whether the *TPM2E122K* mutation affects learning and memory performance in rats, their CA1 areas were randomly transduced with lenti-Flag-vector, lenti-Flag-TPM2WT, or lenti-Flag-TPM2E122K, and the animals were subjected to water maze learning and probe trial tests. Although transduction of lenti-Flag-TPM2WT did not affect spatial acquisition or memory retention, transduction of lenti-Flag-TPM2E122K markedly impaired spatial acquisition compared with the lentivector control and lenti-Flag-TPM2WT groups (Fig. 8c). Transduction of lenti-Flag-TPM2E122K also reduced the time animals spent in the target quadrant during the probe trial test compared with other two groups (Fig. 8d, right panel). A representative swim pattern from each group is also shown (Fig. 8d, upper left panel). Lentivector transduction and expression were confirmed by western blotting with anti-Flag antibody (Fig. 8d, lower-left panel).

## Discussion

Smad4 is a critical effector of TGF-β signaling and is involved in cell differentiation, proliferation, and tumor suppression, but its role in the brain is less clear. This study showed that Smad4 SUMOylation occurs in the brain and facilitates spatial learning and memory formation. In studying the signals upstream of Smad4 SUMOylation, we found that both spatial training and NMDA injection enhanced Smad4 SUMOylation. However, the signaling pathway between NMDA receptor (NMDAR) activation and Smad4 SUMOylation has not yet been determined. We also found that spatial training preferentially and specifically increased the level of Smad3 phosphorylation. NMDAR activation may activate a kinase cascade that initially phosphorylates Smad3 and allows Smad3/Smad4 complex formation, followed by Smad complex translocation to the nucleus. In addition, we previously showed that both spatial training and NMDAR activation increase PIAS1 expression [16, 32]. The presence of increased Smad4 and PIAS1 in the nucleus after training may, therefore, enhance Smad4 SUMOylation. This hypothesis is supported by the finding that TGF-β stimulation induces Smad3 phosphorylation and the formation of the Smad3/Smad4 complex, which binds to the regulatory subunit of protein kinase A (PKA). This cascade mediates TGF-β activation of PKA and CREB for regulation of gene expression [33]. The oncogene forkhead box M1 was found to interact with Smad3 and sustain the Smad3/Smad4 complex, promoting cancer metastasis induced by TGF-β [34]. Although Smad2 and Smad3 are both phosphorylated by TGF-β receptors upon ligand binding, the above results indicate that phosphorylation of Smad3 and the formation of the Smad3/Smad4 complex is sufficient to produce certain biological effects. In contrast, our results do not support the existence of crosstalk between NMDAR signaling and TGF-β signaling. Although Smad3 phosphorylation was increased by both spatial training and TGF-β, Smad2 phosphorylation was markedly reduced by spatial training but increased by TGF-β stimulation [10]. The signaling pathway between NMDAR activation and decreased Smad2 phosphorylation requires further investigation.

In addition to its essential role in neuronal plasticity, NMDA (10 nmol) was found to be moderately excitotoxic to hippocampal neurons [35]. TUNEL assays, however, at the concentration and time intervals used in our study, showed that NMDA was not toxic to CA1 neurons in vivo. One possible explanation for this discrepancy was our use of a lower quantity of NMDA (5.6 nmol) than in the previous study (10 nmol). Alternatively, in the previous study, NMDA was found to be toxic 3 days after injection [35], whereas we tested its effects after 30 and 60 min, intervals that may have been too short to observe any possible excitotoxicity. In addition, comparable CA1 tissue section from each group was used for TUNEL staining. As described previously [32], NMDA at these concentrations did not induce a noticeable increase in the TUNEL signal.

Other than increased Smad3 phosphorylation and decreased Smad2 phosphorylation, we also found that the phosphorylation levels of Smad1 and Smad5 were reduced by spatial training. Bone morphogenetic proteins (BMP) represent the largest subgroup of the TGF-β superfamily [36], and Smad1/Smad5/Smad8 signaling is thought to mediate the biological effects of BMP [25, 37]. Our finding that Smad1 and Smad5 phosphorylation was reduced by spatial training suggests that Smad1 and Smad5 signaling negatively regulates spatial memory formation. This finding is consistent with results showing that transgenic mice with increased BMP signaling show impaired cognition, whereas a BMP inhibitor enhances their cognitive performance [38]. Further studies are required to determine whether there is crosstalk between NMDAR signaling and BMP signaling in the hippocampus and whether activation of BMP signaling decreases Smad4 SUMOylation.

This study also showed that overexpression of Smad4 increased the level of Smad4 SUMOylation but did not facilitate spatial acquisition and memory formation. One possible explanation for this discrepancy is that endogenous Smad4 SUMOylation is not saturated under physiological conditions. Thus, an increase in the amount of Smad4, the substrate protein of PIAS1 E3 ligase, due to overexpression is accompanied by an increase in the level of Smad4 SUMOylation. In contrast, increased Smad4 in cells could form complexes with different phosphorylated Smad proteins, including Smad1, Smad2, Smad3, Smad5, and Smad8. However, our results showed that only phosphorylated Smad3 is associated with spatial acquisition. Therefore, the observed effect of Smad4 overexpression on spatial learning and memory is due to the combined effect of different Smad complexes rather than the Smad3/Smad4 complex alone, with these other Smad complexes impairing spatial memory formation.

In the present study, two Smad4-SUMO bands were observed in some blots but only one Smad4-SUMO band in other blots. This was likely due to differences between overexpression and physiological expression of SUMOylated Smad4. Blots showing two Smad4-SUMO bands were those involving Smad4 plasmid transfection or vector transduction, which markedly increase the SUMOylation signal. In contrast, blots with one Smad4-SUMO band were those involving water maze training or NMDA injection.

This study also showed a low level of Smad4 binding to the *TPM2* promoter in the CA1 area under normal conditions, whereas both RT-PCR and RT-qPCR assays under the same conditions showed high levels of *TPM2* mRNA in the CA1 area. This finding suggested that TPM2 expression in the hippocampus is also regulated by transcription regulators other than the Smad proteins. In addition, like the effect of Smad4 overexpression on spatial acquisition and memory, overexpression of Smad4 was expected to increase the level of Smad4 SUMOylation; however, it decreased *TPM2* mRNA level by about 20%. This result may have been due to the formation of different Smad complexes that can overcome the effects of the Smad3/Smad4 complex alone. Further, transfection of the Smad4 SUMO-mutant almost completely abolished Smad4 binding to the *TPM2* promoter and *TPM2* mRNA expression. The molecular mechanism underlying this effect has not yet been determined. One possibility is that the Lys-113 and Lys-159 residues are located at or near the Smad4 DNA binding sites and mutation of these residues prevents Smad4 DNA binding. A second possibility is that mutations at these two residues can induce a conformational change in Smad4 protein, preventing its binding to DNA. Alternatively, a blockade of Smad4 SUMOylation may result in movement of the Smad3/Smad4 complex away from the Smad4 DNA binding site. In another study, we found that SUMOylation of histone deacetylase 1 (HDAC1) resulted in the movement of the HDAC1 complex away from its DNA binding site [18], suggesting that protein SUMOylation could affect its binding to DNA. Further studies, however, are required to elucidate the detailed mechanism.

TPM2 is predominantly expressed in skeletal muscle, and its function in the brain was unknown. In the present study, we identified a novel role of TPM2 by showing that knockdown of TPM2 expression impairs spatial learning and memory formation. Muscle contraction, which is regulated by TPM2 [39], may be impaired in TPM2 siRNA-transfected animals. The reduced swimming ability of these animals may result in impaired acquisition and memory. However, we found that swimming speed was similar in control siRNA- and TPM2 siRNA-transfected animals, indicating that altered muscle function is not a factor underlying the memory-impairing effect of TPM2 siRNA (Additional file 8). Similar findings were observed following TPM2E122K transduction, because, although the latter impaired spatial learning and memory performance, the swimming speed of these animals was like that of control animals and animals transduced with TPM2WT (Additional file 8). Furthermore, TPM2E122K transduction reduced TPM2 expression by approximately 40% compared with TPM2WT transduction, but it dramatically impaired spatial acquisition. Although TPM2 siRNA transfection decreased TPM2 expression by approximately 75%, the magnitude of spatial acquisition impairment was less than that for TPM2E122K transduction. These observations suggest that TPM2E122K may impair learning and memory through a mechanism other than the downregulation of TPM2 expression. Further investigations are required to determine the molecular mechanisms by which TPM2 siRNA and TPM2E122K impair learning and memory.

## Conclusions

In summary, Smad4 has been regarded primarily as a tumor suppressor downstream of TGF-β signaling, with relatively little known about its role in the nervous system. This study showed that SUMO-modification of Smad4 plays a key role in facilitating spatial learning and memory formation and that *TPM2* is a downstream gene strongly regulated by Smad4 SUMOylation during this process. Further, Smad4 SUMOylation is regulated by NMDAR signaling, which is distinct from TGF-β signaling. Knockdown of TPM2 expression and *TPM2* mutation at E122 both impaired spatial learning and memory performance. Because *TPM2E122K* is one of the mutations identified in patients with skeletal myopathies, this result suggests that patients with certain myopathies may also have deficits in learning and memory functions.

## Methods

### Animals

Adult male Sprague Dawley rats (250–350 g) were used in the present study. All the animals were housed and maintained on a 12/12 h light/dark cycle (light on at 6:30 am) with food and water continuously available. Experimental procedures followed the guidelines and ethical regulations of the Animal Use and Care of the National Institute of Health and were approved by the Animal Committee of the Institute of Biomedical Sciences, Academia Sinica, Taiwan.

### Hippocampal lysate and cell lysate preparation

Animals were killed by decapitation, and their hippocampal tissue was dissected out. Rat hippocampal tissue was lysed by brief sonication in lysis buffer containing 50 mM Tris-HCl (pH 7.4), 150 mM NaCl, 2 mM EDTA, 1% IGEPAL CA-630, 1 mM phenylmethylsulfonyl fluoride (PMSF), 20 μg/ml pepstatin A, 20 μg/ml leupeptin, 20 μg/ml aprotinin, 50 mM NaF, and 1 mM Na_3_VO_4_. HEK293T cell lysate was prepared in 1 ml of lysis buffer containing 20 mM Tris (pH 7.4), 150 mM NaCl, 1 mM MgCl2, 1% IGEPAL CA-630, 10% glycerol, 1 mM dithiothreitol (DTT), 50 mM β-glycerophosphate, 50 mM NaF, 10 μg/ml PMSF, 4 μg/ml aprotinin, 4 μg/ml leupeptin, and 4 μg/ml pepstatin.

### IP and western blot

For IP PIAS1, Smad4, pSmad3, and Flag, the clarified lysate (0.5 mg) was immunoprecipitated with 0.5 μl of anti-PIAS1 antibody (catalog no. 2474-1, Epitomics, Burlingame, CA), 3 μl of anti-Smad4 antibody (catalog no. 9515, Cell Signaling, Danvers, MA), 3 μl of anti-pSmad3 antibody (catalog no. 9520, Cell Signaling), or 2 μl of anti-Flag M2 antibody (catalog no. F1804, Sigma-Aldrich, St. Louis, MO) at 4 °C overnight. Then, 20 μl of rabbit or mouse IgG was used in the control group. The protein A or G magnetic beads (30 ml, 50% slurry, GE Healthcare, Barrington, IL) were added to the IP reaction product to catch the immune complex at 4 °C for 3 h. The immune complex on beads was washed three times with washing buffer containing 20 mM HEPES (pH 7.4), 150 mM NaCl, 1 mM EDTA, 1% IGEPAL CA-630, 1 mM DTT, 50 mM β-glycerophosphate, 50 mM NaF, 10 mg/ml PMSF, 4 μg/ml aprotinin, 4 μg/ml leupeptin, and 4 μg/ml pepstatin and subjected to 8% SDS-PAGE followed by transferring onto the nitrocellulose (NC) membrane (GE Healthcare). Western blot was conducted using the following antibodies: rabbit anti-PIAS1 (1:10000, catalog no. 2474-1, Epitomics), anti-Smad1 (1:3000, catalog no. 6944, Cell Signaling), anti-Smad2 (1:3000, catalog no. 5339, Cell Signaling), anti-Smad3 (1:3000, catalog no. 9523, Cell Signaling), anti-Smad4 (1:3000, catalog no. 9515, Cell Signaling), anti-Smad5 (1:3000, catalog no. 12534, Cell Signaling), anti-pSmad1 (1:2000, catalog no. 5753, Cell Signaling), anti-pSmad2 (1:2000, catalog no. 3108, Cell Signaling), anti-pSmad3 (1:2000, catalog no. 9520, Cell Signaling), anti-pSmad1/5 (1:2000, catalog no. 13820, Cell Signaling), anti-CREB (1:1000, catalog no. 9197, Cell Signaling), anti-pCREB (1:2000, catalog no. 9191, Cell Signaling), anti-SUMO1 (1:4000, catalog no. 40120 SUMOlink kit, Active Motif, Carlsbad, CA), anti-Flag M2 (1:5000, catalog no. F1804, Sigma-Aldrich), anti-enhanced green fluorescent protein (EGFP; 1:5000, catalog no. 11814460001, Roche, Mannheim, Germany), anti-Myc (1:5000, catalog no. 05-419, Millipore, Bedford, MA), anti-His (1:5000, catalog no. OB05, Millipore), anti-GST (1:5000, catalog no. 110736, GeneTex, San Antonio, TX), and anti-actin (1:200000, catalog no. MAB1501, Millipore). The secondary antibody used was horseradish peroxidase (HRP)-conjugated goat-anti rabbit IgG antibody or goat-anti mouse IgG antibody (Chemicon). Membrane was developed by reacting with chemiluminescence HRP substrate (Millipore) and exposed to the LAS-3000 image system (Fujifilm, Tokyo, Japan) for visualization of protein bands. The protein bands were quantified by using the NIH Image J Software (National Institute of Health, MD).

### Plasmid construction and DNA transfection

For construction of the Flag-tagged Smad4 plasmid, full-length *Smad4* was cloned by amplifying the rat hippocampal *Smad4* cDNA (accession NM_019275) with primers 5'-ATCGGAATTCATGGACAATATGTCTATTAC-3' (forward) and 5'-ATCGAAGCTTTCAGTCTAAAGGCTGTGG-3' (reverse). The PCR product was sub-cloned between the EcoRI and HindIII sites of the mammalian expression vector pCMVTag2B. *Smad4* sumo-mutant plasmids were generated using the QuickChange Site-Directed Mutagenesis Kit (Stratagene, La Jolla, CA). For construction of the Flag-tagged *TPM2* plasmid, full-length *TPM2* was cloned by amplifying the rat hippocampal *TPM2* cDNA (accession # NM_001024345) with primers 5'-ATCGGATATCATGGACGCCATCAAGAAG-3' (forward) and 5'-ATATCTCGAGTCAGAGGGAAGTGATGTC-3' (reverse). The PCR product was sub-cloned between the ECoRV and Xho1 sites of the mammalian expression vector pCMVTag2B. For construction of the EGFP-tagged PIAS1 plasmid, full-length *pias1* was sub-cloned into the pEGFP-C1 expression vector (Clontech, Mountain View, CA) with RsrII sites. PIAS1W372A mutant plasmid was generated using the QuickChange Site-Directed Mutagenesis Kit (Stratagene). For construction of the Myc-tagged SUMO1 plasmid, full-length *sumo1* was cloned by amplifying the mouse hippocampal *sumo1* cDNA (accession NM_009460) with primers 5'-GCAACCCGGGTGTCTGACCAGGAGGCAAAACCTTC-3' (forward) and 5'-GCAAGGTACCCTAAACCGTCGAGTGACCCCCCGT-3' (reverse). The PCR product was sub-cloned between the XmaI and KpnI sites of the mammalian expression vector pCMV-Myc. The Myc-SUMO1ΔGG mutant was generated using site-directed mutagenesis [40]. HEK293T cells were maintained in Dulbecco’s modified Eagle’s medium containing 10% fetal bovine serum and incubated at 37 °C in a humidified atmosphere with 5% CO_2_. Transfection was made using the Lipofectamine 2000 reagent (Invitrogen, Carlsbad, CA) in 12-well culture plates according to the manufacturer’s instructions.

### Lentiviral vector construction and preparation

For construction of the Flag-Smad4-tagged lentiviral vectors, full-length Flag-Smad4WT fusion plasmid was sub-cloned into the lentiviral vector pLenti-Tri-cistronic (ABM, Richmond, BC, Canada) by amplifying the construct with the following primers: 5'-ATCGAGTACTGCCACCATGGATTACAAGGATGACGACGATAAGATGGACAATATGTCTATTAC-3' (forward) and 5'-ATCGGGTACCTCAGTCTAAAGGCTGTGG-3' (reverse). The PCR product was sub-cloned between ScaI and KpnI sites of the lentiviral vector. The Smad4 sumo-mutant construct Flag-Smad4K113RK159R was generated using the QuickChange Site-Directed Mutagenesis Kit (Stratagene). For lentivirus packaging, HEK293LTV cells (Cell Biolabs, San Diego, CA) were transfected with 1.5 μg of psPAX2 (Addgene plasmid 12260), 0.5 μg of pMD2.G (Addgene plasmid 12259), and 2 μg of Flag-Smad4WT, 2 μg of Flag-Smad4K113RK159R or 2 μg of pLenti-Tri-cistronic as control using 10 μl of Lipofectamine 2000 (Invitrogen) in a six-well cell culture dish. Lentiviral particles were collected using the speedy lentivirus purification solution (ABM) according to the manufacturer's protocols. A cell culture medium containing lentiviral particles can be harvested two or three times at 12 h intervals until 36 h after transfection. The cell culture was kept at 4 °C for the collecting period. The collected culture medium was further clarified by centrifugation at 2,500 *g* for 10 min and filtrated through a 0.45 μm syringe filter. The speedy lentivirus purification solution (ABM) was added into filtrated supernatant (1:9, v/v) containing lentiviral particles and mixed thoroughly by inversion. The lentiviral supernatant was centrifuged at 5,000 *g* at 4 °C for 10 min. The supernatant was then discarded and the viral pellet was resuspended in ice cold PBS. After titration, the viral stock was stored at -80 °C in aliquots. The lentivirus titer was determined by lentivirus qPCR Titer Kit (ABM) according to the manufacturer's protocols (ABM). The titer of the pLenti-Flag-tagged Smad4WT vector and pLenti-Flag-tagged Smad4K113RK159R vector was 1 × 10^8^ IU/ml.

### In vitro SUMOylation assay

An in vitro SUMOylation assay was performed using the SUMO link kit according to the manufacturer’s instructions (Active Motif, Carlsbad, CA). Recombinant His-tagged Smad4 protein (catalog no. PRO-459, Prospec, East Brunswick, NJ), GST-tagged PIAS1 protein (catalog no. BML-UW9600, Enzo Life Sciences, Ann Arbor, MI), GST-tagged SENP1 protein (catalog no. BML-UW9760-0100, Enzo Life Sciences), 1 μl of E1 activating enzyme, 1 μl of E2 conjugating enzyme, and 0.5 μl of His-SUMO1 protein provided by the kit were added to the SUMOylation buffer. The reaction was carried out at 30 °C for 3 h and the reaction product was then boiled in Laemmli sample buffer at 95 °C for 10 min. The in vitro SUMOylation product was subjected to 8% SDS-PAGE followed by transferring onto the NC membrane. The membrane was immunoblotted with anti-His and anti-GST antibodies.

### Smad4 SUMOylation assay in brain tissue

Hippocampal CA1 tissue lysate was prepared in the same way as that prepared for western blot. For IP Smad4, the clarified lysate (0.5 mg) was immunoprecipitated with 3 μl of anti-Smad4 antibody (catalog no. 9515, Cell Signaling) at 4 °C overnight. The protein A agarose beads (30 ml, 50% slurry, GE Healthcare, Barrington, IL) were added to the IP reaction product to catch the immune complex at 41 °C for 3 h. The immune complex on beads was washed three times with washing buffer containing 20 mM HEPES (pH 7.4), 150 mM NaCl, 1 mM EDTA, 1% IGEPAL CA-630, 1 mM DTT, 50 mM β-glycerophosphate, 50 mM NaF, 10 mg/ml PMSF, 4 mg/ml aprotinin, 4 mg/ml leupeptin, and 4 mg/ml pepstatin and subjected to the SUMOylation reaction with the addition of recombinant PIAS1 protein (3 μl, catalog no. BML-UW9960, Enzo Life Sciences, Farmingdale, NY), E1 (1 μl), E2 (1 μl), and the SUMO1 (0.5 μl) proteins provided in the kit. No de-SUMOylation reagent, such as N-ethyl-maleimide (NEM), was added to the reaction because all tissue lysates were prepared freshly before the SUMOylation assay and Smad4 SUMOylation was readily detectable under this condition. Further, the addition of NEM artificially prevents de-SUMOylation in the cell, which may block the difference in the endogenous SUMOylation signal between groups. The SUMOylation assay was performed using the SUMO linkTM kit according to the manufacturer’s instructions (Active Motif) and boiled in Laemmli sample buffer at 95 °C for 10 min. The SUMOylation product was subjected to 8% SDS-PAGE followed by transferring onto the PVDF membrane (Millipore). The membrane was immunoblotted with anti-Smad4 antibody (1:3000, catalog no. 9515, Cell Signaling) or anti-SUMO1 antibody (1:4000, catalog no. 40120, Active Motif). The remaining procedures were the same as that for carrying out the in vitro SUMOylation assay.

### Smad4 SUMOylation assay in HEK293T cells

For Smad4 SUMOylation determination in HEK293T cells, different Flag-tagged Smad4 plasmids were co-transfected with EGFP-PIAS1 and Myc-SUMO1 plasmids to HEK293T cells. Then 48 h later, the cell lysate was subjected to western blot analyses using anti-Flag (1:5000, catalog no. F1804, Sigma-Aldrich), anti-green fluorescent protein (1:5000, catalog no. 11814460001, Roche, Mannheim, Germany), and anti-Myc (1:5000, catalog no. 05-419, Millipore, Bedford, MA) antibodies.

### Reverse-transcription polymerase chain reaction

RNA was isolated from 20 mg of hippocampal CA1 tissue using RNeasy Mini Kit (Qiagen, Germantown, MD) according to the manufacturer’s instructions. The RNA samples were re-suspended in nuclease-free water and quantified spectrophotometrically at 260 nm. All RNA samples had an A260:A280 value between 1.8 and 2.0. cDNA synthesis was carried out by using the QuantiTect Reverse Transcription Kit (Qiagen) according to the manufacturer’s protocols. The cDNA stock was stored at -20 °C. The hypoxanthine phosphoribosyl transferase (HPRT) mRNA was used as an internal control template. Synthetic primers 5'-CTCTGTGTGCTGAAGGGGGG-3' and 5'-GGGACGCAGCAACAGACATT-3' were used to detect the *HPRT* mRNA, which yielded a PCR product of 625 bp in length. Synthetic primers 5'-AAGGGGACAGAGGATGAG-3' and 5'-CTTTCTCAGCCTCCTCCA-3' were used to detect the *TPM2* mRNA, which yielded a PCR product of 205 bp in length. The PCR reaction was performed according to the following cycle parameters: 94 °C for 2 min followed by 30 cycles at 94 °C for 1 min, 51 °C for 1 min, 72 °C for 1 min, and a final step at 72 °C for 5 min. The PCR product was analyzed on a 1.5% agarose gel (Genemark) and visualized on the EverGene (EverGene Biotechnology) gel analysis system.

### Reverse-transcription quantitative real-time PCR

Total RNA was isolated from 20 mg of hippocampal CA1 tissue using RNeasy Mini Kit (Qiagen, Germantown, MD) according to the manufacturer’s instructions. The RNA samples were resuspended in nuclease-free water and quantified spectrophotometrically at 260 nm. All RNA samples had an *A*260:*A*280 value between 1.8 and 2.0. cDNA synthesis was carried out by using the QuantiTect Reverse Transcription Kit (Qiagen) according to the manufacturer's protocols. The cDNA stock was stored at -20 °C. Quantitative PCR for *TPM2* and the endogenous control gene *HPRT* was carried out using the iQ SYBR Green Supermix (Bio-rad). The primer sequences for *HPRT* were: 5'-GCCGACCGGTTCTGTCAT-3' (forward) and 5'-TCATAACCTGGTTCATCATCACTAATC-3' (reverse). The primer sequences for *TPM2* were: 5'-AAGGGGACAGAGGATGAG-3' (forward) and 5'-CTTTCTCAGCCTCCTCCA-3' (reverse). Amplification was performed using the Rotor-Gene Q Real Time PCR system (Qiagen), and the reaction condition followed the manufacturer’s protocols. The thermal cycler protocol used is as follows: 95 °C for 10 min, 95 °C for 10 s, and 60 °C for 30 s for 40 cycles. The cycle threshold (Ct) values and related data were analyzed using the Rotor-Gene Q Real Time PCR System Software (Qiagen). The expression level of *TPM2* was normalized with that of *HPRT*. The relative expression levels (in fold) were determined using the 2-^(△△Ct)^ method.

### Chromatin immunoprecipitation assay

A chromatin immunoprecipitation (ChIP) assay was performed according to the protocol for the Millipore ChIP assay kit (catalog no. 17-10085). For plasmid DNA transfection, 0.7 μl plasmid DNA complex (1.5 μg/μl) was injected into the rat CA1 area bilaterally 48 h before sacrifice. The CA1 tissue was washed using 1X ice cold PBS and fixed with 1% formaldehyde by adding formaldehyde to the 1X ice cold PBS for 10 min. After adding glycine to quench the unreacted formaldehyde, the tissue was homogenized and resuspended in cell lysis buffer plus protease inhibitor cocktail II, then changed to nuclear lysis buffer plus protease inhibitor cocktail II for sonication. The chromatin was immunoprecipitated using anti-Smad4 antibody (1:3000, catalog no. 9515, Cell Signaling). DNA purified from the immunoprecipitated samples was subjected to the PCR reaction using the following primers for the *TPM2 promoter*. The forward primer is 5'-CAGCCGCAGCTGCCGCTG-3' (nucleotides -432 to -415) and the reverse primer is 5'-ACAAGACCCTTGGGCCGG-3' (nucleotides -363 to -380). The PCR product was 70 bp in length and was separated by agarose gel electrophoresis.

### Intra-hippocampal drug infusion, plasmid DNA transfection, and siRNA injection

Rats were anesthetized with pentobarbital (40 mg/kg) and subjected to stereotaxic surgery. Two 23-gauge stainless-steel thin-wall cannulae were implanted bilaterally to the CA1 area of a rat brain at the following coordinates: 3.5 mm posterior to the bregma, ±2.5 mm lateral to the midline, and 3.4 mm ventral to the skull surface. After recovery from the surgery, NMDA (8 mM) was directly injected to the CA1 area at a rate of 0.1 μl/min. A total of 0.7 μl was injected to each side. For transient Smad4 and TPM2 plasmid DNA transfection, 0.7 μl plasmid DNA complex (1.5 μg/μl) was injected directly to the CA1 area bilaterally in the rat brain using the non-viral transfection agent polyethyleneimine (PEI), and we have previously demonstrated that it does not produce toxicity to hippocampal neurons [41]. Before injection, plasmid DNA was diluted in 5% glucose to a stock concentration of 2.77 μg/μl. Branched PEI of 25 kDa (Sigma-Aldrich) was diluted to 0.1 M concentration in 5% glucose and added to the DNA solution. Immediately before injection, 0.1 M PEI was added to reach a ratio of PEI nitrogen per DNA phosphate equal to 10. The mixture was vortexed for 30 sec and allowed to equilibrate for 15 min. For siRNA injection, 0.7 μl of TPM2 siRNA (8 pmol) or control siRNA was transfected to the CA1 area bilaterally in the rat brain, also using the transfection agent PEI. For the lenti-Smad4-SUMO1 vector and TPM2 siRNA co-injection spatial learning experiment, a sub-threshold concentration of TPM2 siRNA (4 pmol) was used. The sequence for the TPM2 siRNA sense strand is 5′-UCAAACUUCUGGAGGAGAA-3′ and that for the TPM2 siRNA antisense strand is 5′-UUCUCCUCCAGAAGUUUGA-3′. Silencer Negative Control number 1 siRNA was used as a control. They were all synthesized from Ambion®, Thermo Fisher Scientific (Waltham, MA). The inner diameter of the injection needle was 0.31 mm and the wall thickness of the injection needle was 0.12 mm. The injection needle was left in place for 5 min to limit the diffusion of the injected agent. Animals were sacrificed 30 min or 1 h after NMDA (or PBS) injection, and were sacrificed 48 h after plasmid and siRNA transfection. Their brains were removed and cut by a brain slicer. Their CA1 tissue was further punched out using a stainless punch with 2 mm inner diameter. Tissues were frozen at -80 °C until biochemical experimentation.

### Water maze learning

The water maze used was a plastic circular pool, 1.2 m in diameter and 25 cm in height, filled with water (25 ± 2 °C) to a depth of 16 cm. A circular platform of 8 cm in diameter was placed at a specific location away from the edge of the pool. The top of the platform was submerged 0.6 cm below the water surface. The water was made cloudy by adding milk powder. Distinctive visual cues were set on the wall.

For spatial learning, animals were subjected to three trials a day, with one given early in the morning, one given in the early afternoon, and another one given in the late afternoon. The learning procedure lasted for four days and a total of 12 trials were given. For these trials, animals were placed at different starting positions spaced equally around the perimeter of the pool in a random order. Animals were given 60 sec to find the platform. If an animal could not find the platform, it was guided to the platform and was allowed to stay on the platform for 20 sec. The time that each animal took to reach the platform was recorded as the escape latency. A probe trial of 60 sec was given on day 5 to test their memory retention. Animals were placed in the same pool with the platform removed, and the time they spent in each quadrant (target quadrant, left quadrant, opposite quadrant, and right quadrant) was recorded. For the different experiments, the animals were trained for 1 day, 3 days, or 5 days and assigned as the trained animals. Animals that swam for the same period of time in each trial without the presence of the platform and visual cues were assigned as the swim control animals.

### TUNEL staining

TUNEL staining was adopted to detect the apoptotic cells according to the manufacturer’s protocols (Millipore). This is achieved using the Apoptag plus peroxidase in situ apoptosis detection kit. Briefly, brain sections (30-μm thickness) were fixed in 4% paraformaldehyde for 10 min and were permeabilized with pre-cold EtOH/CH3COOH (2:1) for 10 min at -20 °C followed by reacting with 3% H_2_O_2_ for 5 min to remove the endogenous peroxidase activity. The sections were then incubated with the TdT enzyme for 1 h at 37 °C followed by incubation with anti-digoxigenin peroxidase conjugate for 30 min. After washing the specimen with PBS, a 3,3-diaminobenzidine (DAB) peroxidase substrate was applied to the specimen for 3 to 6 min for color development. Apoptotic nuclei became brown with the DAB staining. The slides were then counterstained with methyl blue for visualization of total cells. Cells were examined under a Leica DM IL LED light microscope.

### Statistics

Spatial learning data were analyzed with one-way analysis of variance (ANOVA) or two-way ANOVA with repeated measure followed by post hoc Newman–Keuls multiple comparisons (represented by the *q* value). Biochemical data were analyzed with Student’s *t*-test or one-way ANOVA followed by Newman–Keuls post hoc comparisons. Values of *P* < 0.05 were considered statistically significant (*P < 0.05, **P < 0.01, and ^#^ *P* < 0.001).

## Additional files

Additional file 1: Figure S1. Smad4 is SUMO-modified by PIAS1 in the hippocampus endogenously. **a** Animals were divided into two groups and received control siRNA or PIAS1 siRNA (8 pmol) transfection to their CA1 area. Animals were sacrificed 48 h later and their CA1 tissue was dissected out and subjected to SUMOylation assay without the addition of E1, E1, SUMO1, and the recombinant PIAS1 protein. Left panel: Immunoblotted with anti-Smad4 antibody. Upper right panel: Immunoblotted with anti-SUMO1 antibody. Cell lysate was also subjected to western blot analysis of PIAS1 expression (lower right panel). **b** Quantified results of Smad4 SUMOylation. *n* = 5 each group, *t*(1,8) = 6.1, ^#^ *P* < 0.001. Raw data and statistics are provided as Additional file 8. **c** PIAS1 expression. *n* = 5 each group, *t*(1,8) = 29.31, ^#^ *P* < 0.001. Raw data and statistics are provided as Additional file 8. Data are expressed as mean ± SEM. (PDF 122 kb)

Additional file 2: Figure S2. Spatial training increases the association between PIAS1 and Smad4. Co-IP experiment showing the relationship between PIAS1 and Smad4 in the hippocampus in trained (1 day) and swim control animals. (PDF 51 kb)

Additional file 3: Figure S3. NMDA injection does not produce excitotoxicity to CA1 neurons. PBS, NMDA (8 mM), or kainic acid (0.4 μg) was directly injected into the rat CA1 area and the toxicity to CA1 neurons was examined by TUNEL staining (1 h after PBS and NMDA injection, and 48 h after kainic acid injection). Apoptotic nuclei became brown with 3,3-diaminobenzidine (DAB) peroxidase. The slides were then counterstained with methyl blue for visualization of total cells. Apoptotic cells were observed only under kainic acid treatment, but not NMDA treatment. Scale bar equals 50 μm. Experiments are in duplicate. (PDF 403 kb)

Additional file 4: Figure S4. TGF-β receptor inhibition impairs spatial learning and memory. Animals were divided into two groups and received a PBS or SB525334 (1 μM) injection directly to their CA1 area. They were then subjected to: **a** Water maze learning. *n* = 7 each group, *F*(1,12) = 34.12, ^#^ *P* < 0.001. The statistical difference between the PBS group and SB525334 group for a given trial is indicated by the proper significance sign (*P < 0.05 and ^#^ *P* < 0.001). **b** Probe trial test. *n* = 7 each group, *F*(1,12) = 10.16, **P < 0.01. The representative swim pattern from each group is also shown. Data are expressed as mean ± SEM. PBS phosphate-buffered saline, SEM standard error of the mean (PDF 70 kb)

Additional file 5: Table S1. Genes that are differentially expressed in the CA1 area between Flag-vector and Flag-Smad4K113RK159R-transfected animals. (PDF 127 kb)

Additional file 6: Raw data for Fig. 5b. (PDF 42 kb)

Additional file 7: Raw data for Fig. 5f. (PDF 41 kb)

Additional file 8: Figure S5. Swim speeds of animals with different treatments. (PDF 48 kb)

Additional file 9: Supplementary information. (XLS 62 kb)
