# Supplementary material for: Smad4 SUMOylation is essential for memory formation through upregulation of the skeletal myopathy gene TPM2
Source: BMC Biol. 2017 Nov 28;15:112. doi: 10.1186/s12915-017-0452-9 (PMC5706330; doi:10.1186/s12915-017-0452-9)

Supplementary Figure 1

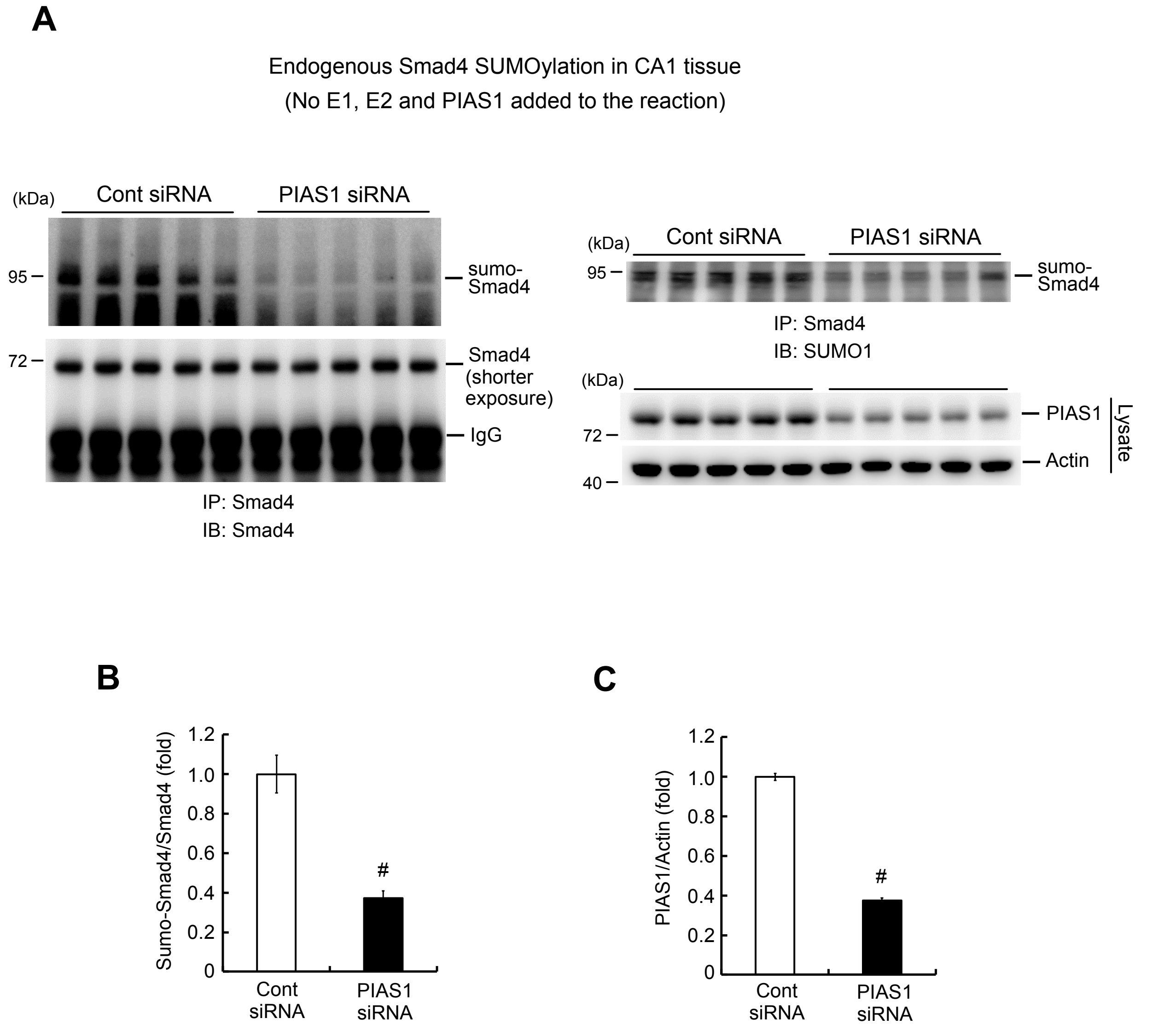

Supplement: Supplementary file 1 — Smad4 is SUMO-modified by PIAS1 in the hippocampus endogenously. a Animals were divided into two groups and received control siRNA or PIAS1 siRNA (8 pmol) transfection to their CA1 area. Animals were sacrificed 48 h later and their CA1 tissue was dissected out and subjected to SUMOylation assay without the addition of E1, E1, SUMO1, and the recombinant PIAS1 protein. Left panel: Immunoblotted with anti-Smad4 antibody. Upper right panel: Immunoblotted with anti-SUMO1 antibody. Cell lysate was also subjected to western blot analysis of PIAS1 expression (lower right panel). b Quantified results of Smad4 SUMOylation. n = 5 each group, t(1,8) = 6.1, # P < 0.001. Raw data and statistics are provided as Additional file 8. c PIAS1 expression. n = 5 each group, t(1,8) = 29.31, # P < 0.001. Raw data and statistics are provided as Additional file 8. Data are expressed as mean ± SEM. (PDF 122 kb) [file 12915_2017_452_MOESM1_ESM.pdf]
