# Supplementary figures and images for: Smad4 SUMOylation is essential for memory formation through upregulation of the skeletal myopathy gene TPM2
Source: BMC Biol. 2017 Nov 28;15:112. doi: 10.1186/s12915-017-0452-9 (PMC5706330; doi:10.1186/s12915-017-0452-9)

# Supplementary Figure 2

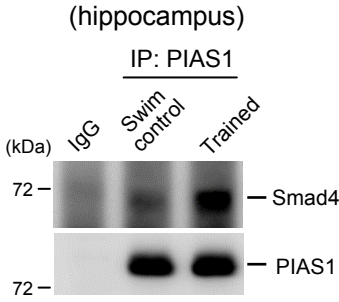

Supplement: Supplementary file 2 — Spatial training increases the association between PIAS1 and Smad4. Co-IP experiment showing the relationship between PIAS1 and Smad4 in the hippocampus in trained (1 day) and swim control animals. (PDF 51 kb) [file 12915_2017_452_MOESM2_ESM.pdf]

## Supplementary Figure 3

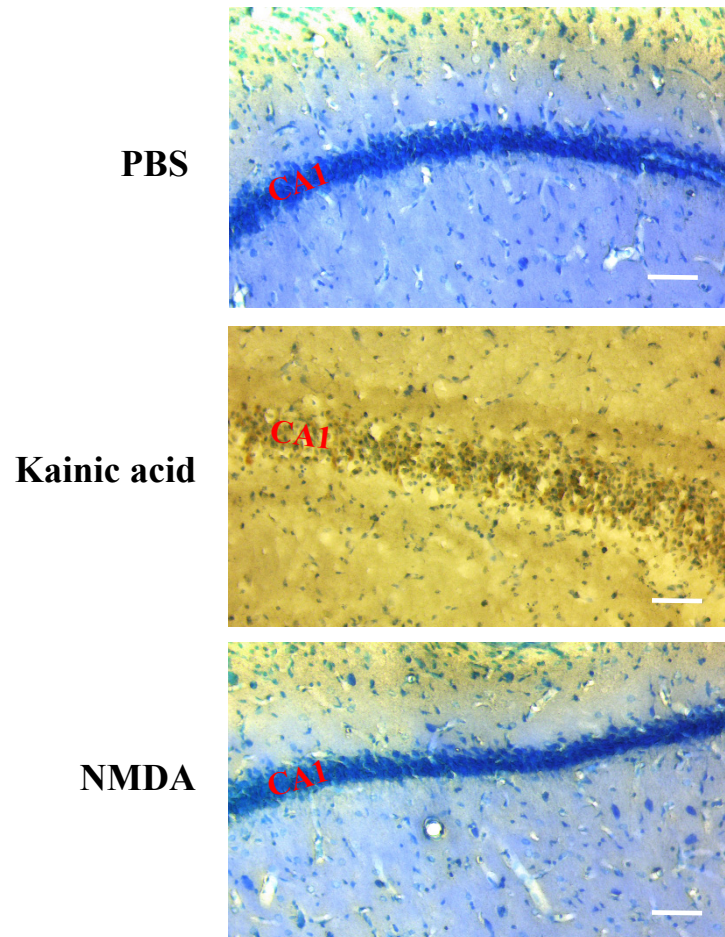

Supplement: Supplementary file 3 — NMDA injection does not produce excitotoxicity to CA1 neurons. PBS, NMDA (8 mM), or kainic acid (0.4 μg) was directly injected into the rat CA1 area and the toxicity to CA1 neurons was examined by TUNEL staining (1 h after PBS and NMDA injection, and 48 h after kainic acid injection). Apoptotic nuclei became brown with 3,3-diaminobenzidine (DAB) peroxidase. The slides were then counterstained with methyl blue for visualization of total cells. Apoptotic cells were observed only under kainic acid treatment, but not NMDA treatment. Scale bar equals 50 μm. Experiments are in duplicate. (PDF 403 kb) [file 12915_2017_452_MOESM3_ESM.pdf]

# Supplementary Figure 4

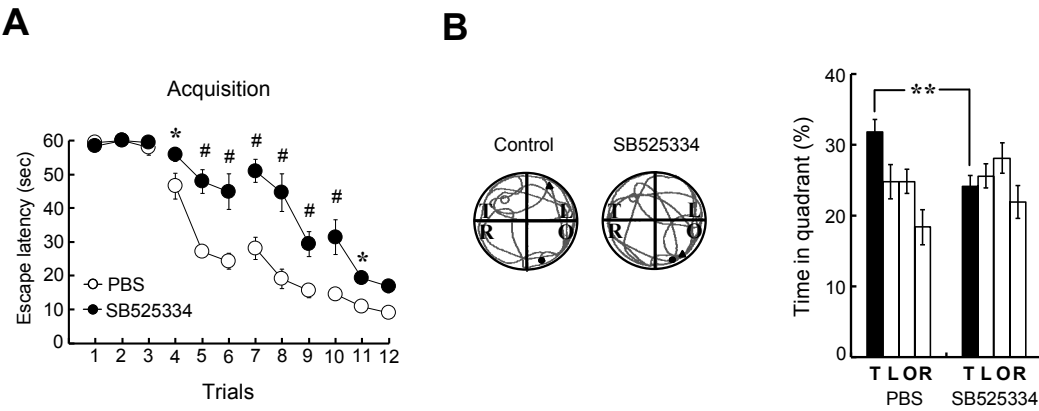

Supplement: Supplementary file 4 — TGF-β receptor inhibition impairs spatial learning and memory. Animals were divided into two groups and received a PBS or SB525334 (1 μM) injection directly to their CA1 area. They were then subjected to: a Water maze learning. n = 7 each group, F(1,12) = 34.12, # P < 0.001. The statistical difference between the PBS group and SB525334 group for a given trial is indicated by the proper significance sign (*P < 0.05 and # P < 0.001). b Probe trial test. n = 7 each group, F(1,12) = 10.16, **P < 0.01. The representative swim pattern from each group is also shown. Data are expressed as mean ± SEM. PBS phosphate-buffered saline, SEM standard error of the mean (PDF 70 kb) [file 12915_2017_452_MOESM4_ESM.pdf]
