# Supplementary material for: Smad4 SUMOylation is essential for memory formation through upregulation of the skeletal myopathy gene TPM2
Source: BMC Biol. 2017 Nov 28;15:112. doi: 10.1186/s12915-017-0452-9 (PMC5706330; doi:10.1186/s12915-017-0452-9)
Supplement: Supplementary file 5 — Genes that are differentially expressed in the CA1 area between Flag-vector and Flag-Smad4K113RK159R-transfected animals. (PDF 127 kb) [file 12915_2017_452_MOESM5_ESM.pdf]

Table S1. Genes that are differentially expressed in the CA1 area between Flag-vector and Flag-Smad4K113RK159R-transfected animals

GenbankAccession	Gene	Description	Fold	P value
1. Extracellular component and cell adhesion (extra cellular matrix and structure organiz)				
NM_001108254	EGFL6	Rattus norvegicus EGF-like-domain, multiple 6 (Egfl6), mRNA [NM_001108254]	2.27759	9.63E-03
NM_134336	NLGN3	Rattus norvegicus neuroligin 3 (Nlgn3), mRNA [NM_134336]	-2.1648147	1.09E-02
U11038	LOX	Rattus norvegicus lysyl oxidase mRNA, complete cds and multiple polyadenylation sites. [U11038]	-2.1971967	9.63E-03
XM_213954	NID1	Nidogen-1 [Source:UniProtKB/Swiss-Prot;Acc:P08460] [ENSRNOT00000003349]	-2.3879435	9.63E-03
NM_053489	COL18A1	Rattus norvegicus collagen, type XVIII, alpha 1 (Col18a1), mRNA [NM_053489]	-2.5567176	1.09E-02
NM_138518	CRISPLD2	Rattus norvegicus cysteine-rich secretory protein LCCL domain containing 2 (Crispld2), mRNA [NM_138518]	-2.5611393	9.63E-03
AF195789	DRP2	Rattus norvegicus dystrophin-related protein 2 splice variant (Drp2) mRNA, partial cds. [AF195789]	-3.3624563	1.09E-02
NM_032085	COL3A1	Rattus norvegicus collagen, type III, alpha 1 (Col3a1), mRNA [NM_032085]	-3.8684728	9.63E-03
NM_001170600	MYH11	Rattus norvegicus myosin, heavy chain 11, smooth muscle (Myh11), mRNA [NM_001170600]	-4.666121	1.09E-02
NM_053304	COL1A1	Rattus norvegicus collagen, type I, alpha 1 (Col1a1), mRNA [NM_053304]	-4.968433	1.09E-02
NM_053356	COL1A2	Rattus norvegicus collagen, type I, alpha 2 (Col1a2), mRNA [NM_053356]	-5.3541346	9.63E-03
2. Response to steroid hormone stimuli				
NM_173331	MAPK15	Rattus norvegicus mitogen-activated protein kinase 15 (Mapk15), mRNA [NM_173331]	2.507982	5.29E-02
NM_080782	CDKN1A	Rattus norvegicus cyclin-dependent kinase inhibitor 1A (Cdkn1a), mRNA [NM_080782]	2.218104	5.29E-02
M89906	CFTR	Rattus norvegicus cystic fibrosis transmembrane conductance regulator (CFTR) gene, partial cds. [M89906]	-2.25299	5.29E-02
NM_012778	AQP1	Rattus norvegicus aquaporin 1 (Aqp1), mRNA [NM_012778]	-2.36801	1.84E-02
NM_053566	PTCH1	Rattus norvegicus patched 1 (Ptch1), mRNA [NM_053566]	-2.42153	5.29E-02
NM_012490	ACR	Rattus norvegicus acrosin (Acr), mRNA [NM_012490]	-2.4797	5.29E-02
NM_021669	GHRL	Rattus norvegicus ghrelin/obestatin prepropeptide (Ghrl), mRNA [NM_021669]	-2.60403	5.29E-02
NM_031511	IGF2	Rattus norvegicus insulin-like growth factor 2 (Igf2), transcript variant 1, mRNA [NM_031511]	-3.08452	5.29E-02
NM_053896	ALDH1A2	Rattus norvegicus aldehyde dehydrogenase 1 family, member A2 (Aldh1a2), mRNA [NM_053896]	-3.59086	5.29E-02
NM_031650	SLCO1B3	Rattus norvegicus solute carrier organic anion transporter family, member 1b3 (Slco1b3), transcript variant 1, mRNA [NM_031650]	-3.69145	8.30E-03
NM_013015	PTGDS	Rattus norvegicus prostaglandin D2 synthase (brain) (Ptgds), mRNA [NM_013015]	-3.92076	5.29E-02
3. Embryonic morphogenesis				
NM_017144	TNNI3	Rattus norvegicus troponin I type 3 (cardiac) (Tnni3), mRNA [NM_017144]	3.150758	2.04E-02
NM_019282	GREM1	Rattus norvegicus gremlin 1 (Grem1), mRNA [NM_019282]	2.055063	5.33E-03
NM_080778	NR2F2	Rattus norvegicus nuclear receptor subfamily 2, group F, member 2 (Nr2f2), mRNA [NM_080778]	-2.03074	2.04E-02
NM_012550	EDNRA	Rattus norvegicus endothelin receptor type A (Ednra), mRNA [NM_012550]	-2.05533	2.04E-02
NM_017087	BGN	Rattus norvegicus biglycan (Bgn), mRNA [NM_017087]	-2.08882	2.04E-02
NM_138851	PROK1	Rattus norvegicus prokineticin 1 (Prok1), mRNA [NM_138851]	-2.0987	2.04E-02
BC097327	HAND1	Rattus norvegicus heart and neural crest derivatives expressed 1, mRNA (cDNA clone MGC:114332 IMAGE:7444552), complete cds. [BC097327]	-2.12664	2.04E-02

NM_001106519	OVOL2	Rattus norvegicus ovo-like 2 (Drosophila) (Ovol2), mRNA [NM_001106519]	-2.36322	2.04E-02
NM_181375	ROBO4	Rattus norvegicus roundabout homolog 4 (Drosophila) (Robo4), mRNA [NM_181375]	-2.76545	2.04E-02
NM_053530	TWIST1	Rattus norvegicus twist basic helix-loop-helix transcription factor 1 (Twist1), mRNA [NM_053530]	-2.77763	7.92E-03
NM_017244	CRABP2	Rattus norvegicus cellular retinoic acid binding protein 2 (Crabp2), mRNA [NM_017244]	-3.86889	1.31E-02
NM_181368	MUSTN1	Rattus norvegicus musculoskeletal, embryonic nuclear protein 1 (Mustn1), mRNA [NM_181368]	-4.88589	6.62E-03
NM_001007012	ALX3	Rattus norvegicus aristaless-like homeobox 3 (Alx3), mRNA [NM_001007012]	-6.43535	5.33E-03

4. Skeletal system

NM_053802	TGFBI	Rattus norvegicus transforming growth factor, beta induced (Tgfbi), mRNA [NM_053802]	2.171796	7.51E-02
NM_052803	ATP7A	Rattus norvegicus ATPase, Cu++ transporting, alpha polypeptide (Atp7a), mRNA [NM_052803]	2.12499	7.51E-02
NM_012918	CACNA1A	Rattus norvegicus calcium channel, voltage-dependent, P/Q type, alpha 1A subunit (Cacna1a), mRNA [NM_012918]	2.095481	8.00E-02
NM_001106591	ATP6V0A4	Rattus norvegicus ATPase, H+ transporting, lysosomal V0 subunit A4 (Atp6v0a4), mRNA [NM_001106591]	-2.07675	7.51E-02
NM_001105884	HOXD1	Rattus norvegicus homeo box D1 (Hoxd1), mRNA [NM_001105884]	-2.09308	7.51E-02
NM_001107034	TBX4	Rattus norvegicus T-box 4 (Tbx4), mRNA [NM_001107034]	-2.15903	7.51E-02
NM_001173469	HOXD9	Rattus norvegicus homeo box D9 (Hoxd9), mRNA [NM_001173469]	-2.21485	7.51E-02
NM_001109884	HOXC4	Rattus norvegicus homeo box C4 (Hoxc4), mRNA [NM_001109884]	-2.25763	7.51E-02
NM_017140	DRD3	Rattus norvegicus dopamine receptor D3 (Drd3), mRNA [NM_017140]	-2.25936	8.00E-02
NM_001105792	HES7	Rattus norvegicus hairy and enhancer of split 7 (Drosophila) (Hes7), mRNA [NM_001105792]	-2.30159	7.51E-02
NM_012862	MGP	Rattus norvegicus matrix Gla protein (Mgp), mRNA [NM_012862]	-2.32403	7.51E-02
NM_138881	RSAD2	Rattus norvegicus radical S-adenosyl methionine domain containing 2 (Rsad2), mRNA [NM_138881]	-4.40908	7.51E-02
NM_001134604	CYTL1	Rattus norvegicus cytokine like 1 (Cytl1), mRNA [NM_001134604]	-5.90325	7.51E-02
NM_001024345	TPM2	Rattus norvegicus tropomyosin 2, beta (Tpm2), mRNA [NM_001024345]	-8.13361	8.00E-02

5. Wounding healing and defense response

X76129	PLAUR	Urokinase plasminogen activator surface receptor [Source:UniProtKB/Swiss-Prot;Acc:P49616] [ENSRNOT00000018222]	3.645878	1.20E-02
XM_001058477	DSP	Uncharacterized protein [Source:UniProtKB/TrEMBL;Acc:F1LMV6] [ENSRNOT00000018649]	3.226811	2.44E-03
XM_001066203	VWF	von Willebrand factor [Source:UniProtKB/Swiss-Prot;Acc:Q62935] [ENSRNOT00000026643]	-2.06093	1.20E-02
NM_001079898	RATNP-3B	Rattus norvegicus defensin RatNP-3 precursor (RatNP-3b), mRNA [NM_001079898]	-2.09174	3.80E-02
NM_019630	GIP	Rattus norvegicus gastric inhibitory polypeptide (Gip), mRNA [NM_019630]	-2.20526	2.85E-02
NM_173299	NP4	Rattus norvegicus defensin NP-4 precursor (Np4), mRNA [NM_173299]	-2.3707	3.80E-02
NM_134391	DEFB22	Rattus norvegicus defensin beta 22 (Defb22), mRNA [NM_134391]	-2.58869	3.80E-02
NM_001109641	HIST3H2BB	Rattus norvegicus histone cluster 3, H2bb (Hist3h2bb), mRNA [NM_001109641]	-2.7433	3.80E-02

6. Inflammation and immune response

NM_001004084	RT1-BB	Rattus norvegicus RT1 class II, locus Bb (RT1-Bb), mRNA [NM_001004084]	857.6616	9.63E-07
X14879	RT1-BA	Rat mRNA for RT1.B-1(alpha) chain of integral membrane protein. [X14879]	198.1676	9.63E-07

XM_003751489	MPHOSPH8	M-phase phosphoprotein 8 [Source:RefSeq peptide;Acc:NP_001017375] [ENSRNOT00000049420]	24.81877	6.16E-02
NM_053647	CXCL2	Rattus norvegicus chemokine (C-X-C motif) ligand 2 (Cxcl2), mRNA [NM_053647]	5.968048	6.16E-02
NM_138522	CXCL3	Rattus norvegicus chemokine (C-X-C motif) ligand 3 (Cxcl3), mRNA [NM_138522]	5.529883	6.16E-02
AF068268	OAS1B	2-5 oligoadenylate synthetase 1B [Source:RefSeq peptide;Acc:NP_653353] [ENSRNOT00000001853]	5.446364	8.41E-05
NM_013025	CCL3	Rattus norvegicus chemokine (C-C motif) ligand 3 (Ccl3), mRNA [NM_013025]	4.272974	6.16E-02
NM_001037357	LILRB3L	Rattus norvegicus leukocyte immunoglobulin-like receptor, subfamily B (with TM and ITIM domains), member 3-like (Lilrb3l), mRNA [NM_001037357]	3.85408	9.63E-07
NM_001003707	CLEC4D	Rattus norvegicus C-type lectin domain family 4, member D (Clec4d), mRNA [NM_001003707]	3.318973	9.63E-07
NM_017019	IL1A	Rattus norvegicus interleukin 1 alpha (Il1a), mRNA [NM_017019]	3.092943	6.16E-02
NM_020542	CCR1	Rattus norvegicus chemokine (C-C motif) receptor 1 (Ccr1), mRNA [NM_020542]	2.876091	6.16E-02
NM_022194	IL1RN	Rattus norvegicus interleukin 1 receptor antagonist (Il1rn), mRNA [NM_022194]	2.147939	6.16E-02
NM_130426	TNFRSF1B	Rattus norvegicus tumor necrosis factor receptor superfamily, member 1b (Tnfrsf1b), mRNA [NM_130426]	2.139479	6.16E-02
NM_001007729	PF4	Rattus norvegicus platelet factor 4 (Pf4), mRNA [NM_001007729]	2.114858	9.63E-07
NM_031713	LILRB3	Rattus norvegicus leukocyte immunoglobulin-like receptor, subfamily B (with TM and ITIM domains), member 3 (Lilrb3), mRNA [NM_031713]	2.046807	9.63E-07
NM_019130	INS2	Rattus norvegicus insulin 2 (Ins2), mRNA [NM_019130]	-2.02176	6.16E-02
NM_001008855	RT1-N3	Rattus norvegicus RT1 class Ib, locus N3 (RT1-N3), mRNA [NM_001008855]	-2.02832	9.63E-07
XM_001076062	IFNA2	Uncharacterized protein [Source:UniProtKB/TrEMBL;Acc:D3ZLZ2] [ENSRNOT00000046931]	-2.03814	8.41E-05
NM_001008886	RT1-S3	Rattus norvegicus RT1 class Ib, locus S3 (RT1-S3), mRNA [NM_001008886]	-2.05362	9.63E-07
NM_019335	EIF2AK2	Rattus norvegicus eukaryotic translation initiation factor 2-alpha kinase 2 (Eif2ak2), mRNA [NM_019335]	-2.07675	8.41E-05
NM_017208	LBP	Rattus norvegicus lipopolysaccharide binding protein (Lbp), mRNA [NM_017208]	-2.08724	6.16E-02
NM_001002821	RT1-T18	Rattus norvegicus RT1 class Ib, locus T18 (RT1-T18), mRNA [NM_001002821]	-2.10324	9.63E-07
NM_057146	C9	Rattus norvegicus complement component 9 (C9), mRNA [NM_057146]	-2.14707	6.16E-02
NM_012737	APOA4	Rattus norvegicus apolipoprotein A-IV (Apoa4), mRNA [NM_012737]	-2.19934	9.63E-07
NM_145672	CXCL9	Rattus norvegicus chemokine (C-X-C motif) ligand 9 (Cxcl9), mRNA [NM_145672]	-2.23044	9.63E-07
NM_012848	FTH1	Rattus norvegicus ferritin, heavy polypeptide 1 (Fth1), mRNA [NM_012848]	-2.27844	9.63E-07
NM_001191743	SAMHD1	Rattus norvegicus SAM domain and HD domain, 1 (Samhd1), mRNA [NM_001191743]	-2.29442	8.41E-05
NM_016994	C3	Rattus norvegicus complement component 3 (C3), mRNA [NM_016994]	-2.31722	6.16E-02
NM_013049	TNFRSF4	Rattus norvegicus tumor necrosis factor receptor superfamily, member 4 (Tnfrsf4), mRNA [NM_013049]	-2.33135	6.16E-02
NM_001105962	PRG4	Rattus norvegicus proteoglycan 4, (megakaryocyte stimulating factor, articular superficial zone protein, camptodactyly, arthropathy, coxa vara, pericarditis syndrome) (Prg4), mRNA [NM_001105962]	-2.35979	9.63E-07
NM_057203	CCL22	Rattus norvegicus chemokine (C-C motif) ligand 22 (Ccl22), mRNA [NM_057203]	-2.36569	9.63E-07
NM_012646	RT1-N1	Rattus norvegicus RT1 class Ib, locus N1 (RT1-N1), mRNA [NM_012646]	-2.38438	9.63E-07
NM_001009682	OASL2	Rattus norvegicus 2'-5' oligoadenylate synthetase-like 2 (Oasl2), mRNA [NM_001009682]	-2.57308	9.63E-07

NM_199082	SECTM1B	Rattus norvegicus secreted and transmembrane 1B (Sectm1b), mRNA [NM_199082]	-2.57443	9.63E-07
NM_001008510	ISG20	Rattus norvegicus interferon stimulated exonuclease gene 20 (Isg20), mRNA [NM_001008510]	-2.65899	8.41E-05
NM_001011931	MILL1	Rattus norvegicus MHC I like leukocyte 1 (Mill1), mRNA [NM_001011931]	-2.72101	9.63E-07
NM_001017496	CXCL13	Rattus norvegicus chemokine (C-X-C motif) ligand 13 (Cxcl13), mRNA [NM_001017496]	-2.86759	9.63E-07
NM_182952	CXCL11	Rattus norvegicus chemokine (C-X-C motif) ligand 11 (Cxcl11), mRNA [NM_182952]	-3.16223	9.63E-07
NM_031513	IL3	Rattus norvegicus interleukin 3 (Il3), mRNA [NM_031513]	-3.58277	9.63E-07
NM_001033691	IRF7	Rattus norvegicus interferon regulatory factor 7 (Irf7), mRNA [NM_001033691]	-4.01603	8.41E-05
NM_138913	OAS1A	Rattus norvegicus 2'-5' oligoadenylate synthetase 1A (Oas1a), mRNA [NM_138913]	-4.24098	8.41E-05
NM_001009680	OAS1I	Rattus norvegicus 2 ' -5 ' oligoadenylate synthetase 1I (Oas1i), mRNA [NM_001009680]	-4.55671	9.63E-07
NM_173096	MX1	Rattus norvegicus myxovirus (influenza virus) resistance 1 (Mx1), transcript variant 1, mRNA [NM_173096]	-4.56277	8.41E-05
NM_139089	CXCL10	Rattus norvegicus chemokine (C-X-C motif) ligand 10 (Cxcl10), mRNA [NM_139089]	-4.71462	8.41E-05
NM_001008833	RT1-CE10	Rattus norvegicus RT1 class I, locus CE10 (RT1-CE10), mRNA [NM_001008833]	-6.27704	9.63E-07
NM_001008884	RT1-DB1	Rattus norvegicus RT1 class II, locus Db1 (RT1-Db1), mRNA [NM_001008884]	-8.04773	9.63E-07
NM_053289	REG3B	Rattus norvegicus regenerating islet-derived 3 beta (Reg3b), mRNA [NM_053289]	-10.6161	6.16E-02
NM_031810	DEFB1	Rattus norvegicus defensin beta 1 (Defb1), mRNA [NM_031810]	-19.571	9.63E-07

7. Protein kinase cascade and signal transduction

NM_001100700	SFRP2	Rattus norvegicus secreted frizzled-related protein 2 (Sfrp2), mRNA [NM_001100700]	8.909609	4.56E-07
NM_012611	NOS2	Rattus norvegicus nitric oxide synthase 2, inducible (Nos2), mRNA [NM_012611]	4.676464	4.56E-07
NM_001001272	OLR130	Rattus norvegicus olfactory receptor 130 (Olr130), mRNA [NM_001001272]	3.137423	4.56E-07
NM_001000656	OLR604	Rattus norvegicus olfactory receptor 604 (Olr604), mRNA [NM_001000656]	3.079256	4.56E-07
NM_001000739	OLR150	Rattus norvegicus olfactory receptor 150 (Olr150), mRNA [NM_001000739]	2.875733	4.56E-07
NM_053688	PDE6H	Rattus norvegicus phosphodiesterase 6H, cGMP-specific, cone, gamma (Pde6h), mRNA [NM_053688]	2.638865	2.49E-02
NM_001000070	OLR1016	Rattus norvegicus olfactory receptor 1016 (Olr1016), mRNA [NM_001000070]	2.621737	4.56E-07
NM_001135033	TRIM16	Rattus norvegicus tripartite motif-containing 16 (Trim16), mRNA [NM_001135033]	2.547059	2.49E-02
NM_001000715	OLR1501	Rattus norvegicus olfactory receptor 1501 (Olr1501), mRNA [NM_001000715]	2.54658	4.56E-07
NM_001000400	OLR851	Rattus norvegicus olfactory receptor 851 (Olr851), mRNA [NM_001000400]	2.439552	4.56E-07
NM_080407	BMPR2	Rattus norvegicus bone morphogenetic protein receptor, type II (serine/threonine kinase) (Bmpr2), mRNA [NM_080407]	2.439391	4.56E-07
NM_001000083	OLR1589	Rattus norvegicus olfactory receptor 1589 (Olr1589), mRNA [NM_001000083]	2.405317	4.56E-07
NM_001005557	AKAP3	Rattus norvegicus A kinase (PRKA) anchor protein 3 (Akap3), mRNA [NM_001005557]	2.328926	4.56E-07
NM_001000821	OLR868	Rattus norvegicus olfactory receptor 868 (Olr868), mRNA [NM_001000821]	2.242694	4.56E-07

NM_017250	HTR2B	Rattus norvegicus 5-hydroxytryptamine (serotonin) receptor 2B, G protein-coupled (Htr2b), mRNA [NM_017250]	2.228374	2.49E-02
NM_001108444	GAB1	Rattus norvegicus GRB2-associated binding protein 1 (Gab1), mRNA [NM_001108444]	2.200933	4.60E-02
NM_001000525	OLR1433	Rattus norvegicus olfactory receptor 1433 (Olr1433), mRNA [NM_001000525]	2.175105	4.56E-07
NM_001000934	OLR463	Rattus norvegicus olfactory receptor 463 (Olr463), mRNA [NM_001000934]	2.146127	4.56E-07
NM_175587	TAAR7H	Rattus norvegicus trace amine-associated receptor 7h (Taar7h), mRNA [NM_175587]	2.135173	4.56E-07
NM_001000804	OLR1261	Rattus norvegicus olfactory receptor 1261 (Olr1261), mRNA [NM_001000804]	2.128192	4.56E-07
NM_182953	NEK6	Rattus norvegicus NIMA-related kinase 6 (Nek6), mRNA [NM_182953]	2.018253	2.49E-02
NM_053429	FGFR3	Rattus norvegicus fibroblast growth factor receptor 3 (Fgfr3), mRNA [NM_053429]	-2.0026	4.60E-02
NM_001000630	OLR691	Rattus norvegicus olfactory receptor 691 (Olr691), mRNA [NM_001000630]	-2.01254	4.56E-07
NM_153720	ITGB3	Rattus norvegicus integrin, beta 3 (Itgb3), mRNA [NM_153720]	-2.01422	4.60E-02
NM_001000040	OLR1522	Rattus norvegicus olfactory receptor 1522 (Olr1522), mRNA [NM_001000040]	-2.02112	4.56E-07
NM_001013915	TAS2R134	Rattus norvegicus taste receptor, type 2, member 134 (Tas2r134), mRNA [NM_001013915]	-2.03226	4.56E-07
NM_001000837	OLR1631	Rattus norvegicus olfactory receptor 1631 (Olr1631), mRNA [NM_001000837]	-2.03329	4.56E-07
NM_001001092	OLR1395	Rattus norvegicus olfactory receptor 1395 (Olr1395), mRNA [NM_001001092]	-2.03575	4.56E-07
NM_001000668	OLR559	Rattus norvegicus olfactory receptor 559 (Olr559), mRNA [NM_001000668]	-2.0383	4.56E-07
NM_001000128	OLR39	Rattus norvegicus olfactory receptor 39 (Olr39), mRNA [NM_001000128]	-2.04365	4.56E-07
NM_001106447	PYGO2	Rattus norvegicus pygopus 2 (Pygo2), mRNA [NM_001106447]	-2.04435	4.56E-07
NM_001000356	OLR687	Rattus norvegicus olfactory receptor 687 (Olr687), mRNA [NM_001000356]	-2.0461	4.56E-07
NM_001000610	OLR771	Rattus norvegicus olfactory receptor 771 (Olr771), mRNA [NM_001000610]	-2.04873	4.56E-07
NM_001000642	OLR646	Rattus norvegicus olfactory receptor 646 (Olr646), mRNA [NM_001000642]	-2.05226	4.56E-07
NM_031804	CISH	Rattus norvegicus cytokine inducible SH2-containing protein (Cish), mRNA [NM_031804]	-2.05475	4.56E-07
NM_001000245	OLR323	Rattus norvegicus olfactory receptor 323 (Olr323), mRNA [NM_001000245]	-2.05557	4.56E-07
NM_001000306	OLR484	Rattus norvegicus olfactory receptor 484 (Olr484), mRNA [NM_001000306]	-2.05684	4.56E-07
NM_001034920	CBLC	Rattus norvegicus Cbl proto-oncogene, E3 ubiquitin protein ligase C (Cblc), mRNA [NM_001034920]	-2.05928	4.56E-07
NM_001000317	OLR517	Rattus norvegicus olfactory receptor 517 (Olr517), mRNA [NM_001000317]	-2.05992	4.56E-07
NM_001000519	OLR1307	Rattus norvegicus olfactory receptor 1307 (Olr1307), mRNA [NM_001000519]	-2.07275	4.56E-07
NM_001000231	OLR288	Rattus norvegicus olfactory receptor 288 (Olr288), mRNA [NM_001000231]	-2.07962	4.56E-07
NM_032612	STAT1	Rattus norvegicus signal transducer and activator of transcription 1 (Stat1), transcript variant alpha, mRNA [NM_032612]	-2.08293	4.56E-07
NM_001012107	GPR157	Rattus norvegicus G protein-coupled receptor 157 (Gpr157), mRNA [NM_001012107]	-2.08395	4.56E-07
NM_001000242	OLR309	Rattus norvegicus olfactory receptor 309 (Olr309), mRNA [NM_001000242]	-2.08608	4.56E-07
NM_001001283	OLR137	Rattus norvegicus olfactory receptor 137 (Olr137), mRNA [NM_001001283]	-2.08735	4.56E-07

NM_153727	GPR3	Rattus norvegicus G protein-coupled receptor 3 (Gpr3), mRNA [NM_153727]	-2.10276	4.56E-07
NM_175586	TAAR7B	Rattus norvegicus trace amine-associated receptor 7b (Taar7b), mRNA [NM_175586]	-2.10469	4.56E-07
NM_001000266	OLR1671	Rattus norvegicus olfactory receptor 1671 (Olr1671), mRNA [NM_001000266]	-2.10938	4.56E-07
NM_001099508	VOM2R61	Rattus norvegicus vomeronasal 2 receptor, 61 (Vom2r61), mRNA [NM_001099508]	-2.11311	4.56E-07
NM_001000387	OLR416	Rattus norvegicus olfactory receptor 416 (Olr416), mRNA [NM_001000387]	-2.11587	4.56E-07
NM_133573	GPER	Rattus norvegicus G protein-coupled estrogen receptor 1 (Gper), mRNA [NM_133573]	-2.12338	4.56E-07
NM_013108	ADRB3	Rattus norvegicus adrenoceptor beta 3 (Adrb3), mRNA [NM_013108]	-2.1292	2.49E-02
NM_001000125	OLR44	Rattus norvegicus olfactory receptor 44 (Olr44), mRNA [NM_001000125]	-2.13276	4.56E-07
NM_020106	OLR414	Rattus norvegicus olfactory receptor 414 (Olr414), mRNA [NM_020106]	-2.13845	4.56E-07
NM_001107133	GIGYF1	Rattus norvegicus GRB10 interacting GYF protein 1 (Gigyf1), mRNA [NM_001107133]	-2.14857	4.56E-07
NM_031766	CPZ	Rattus norvegicus carboxypeptidase Z (Cpz), mRNA [NM_031766]	-2.15674	4.56E-07
NM_001000199	OLR217	Rattus norvegicus olfactory receptor 217 (Olr217), mRNA [NM_001000199]	-2.16935	4.56E-07
NM_001000611	OLR767	Rattus norvegicus olfactory receptor 767 (Olr767), mRNA [NM_001000611]	-2.17199	4.56E-07
NM_001000623	OLR715	Rattus norvegicus olfactory receptor 715 (Olr715), mRNA [NM_001000623]	-2.17741	4.56E-07
NM_012882	SSTR5	Rattus norvegicus somatostatin receptor 5 (Sstr5), mRNA [NM_012882]	-2.18204	4.56E-07
NM_001107410	GPR114	Rattus norvegicus G protein-coupled receptor 114 (Gpr114), mRNA [NM_001107410]	-2.1969	4.56E-07
NM_001000705	OLR922	Rattus norvegicus olfactory receptor 922 (Olr922), mRNA [NM_001000705]	-2.20206	4.56E-07
NM_001000155	OLR121	Rattus norvegicus olfactory receptor 121 (Olr121), mRNA [NM_001000155]	-2.21159	4.56E-07
NM_001000465	OLR1306	Rattus norvegicus olfactory receptor 1306 (Olr1306), mRNA [NM_001000465]	-2.23926	4.56E-07
NM_001100641	MTNR1B	Rattus norvegicus melatonin receptor 1B (Mtnr1b), mRNA [NM_001100641]	-2.23955	4.56E-07
NM_001000447	OLR1235	Rattus norvegicus olfactory receptor 1235 (Olr1235), mRNA [NM_001000447]	-2.25177	4.56E-07
NM_001000577	OLR744	Rattus norvegicus olfactory receptor 744 (Olr744), mRNA [NM_001000577]	-2.25249	4.56E-07
NM_001000016	OLR1439	Rattus norvegicus olfactory receptor 1439 (Olr1439), mRNA [NM_001000016]	-2.25286	4.56E-07
NM_001000003	OLR1401	Rattus norvegicus olfactory receptor 1401 (Olr1401), mRNA [NM_001000003]	-2.26749	4.56E-07
NM_001000675	OLR527	Rattus norvegicus olfactory receptor 527 (Olr527), mRNA [NM_001000675]	-2.26774	4.56E-07
NM_001001098	OLR1515	Rattus norvegicus olfactory receptor 1515 (Olr1515), mRNA [NM_001001098]	-2.26965	4.56E-07
NM_001000700	OLR996	Rattus norvegicus olfactory receptor 996 (Olr996), mRNA [NM_001000700]	-2.28415	4.56E-07
NM_001000980	OLR1366	Rattus norvegicus olfactory receptor 1366 (Olr1366), mRNA [NM_001000980]	-2.28522	4.56E-07
NM_053882	ECM1	Rattus norvegicus extracellular matrix protein 1 (Ecm1), mRNA [NM_053882]	-2.30377	2.49E-02
NM_001000221	OLR262	Rattus norvegicus olfactory receptor 262 (Olr262), mRNA [NM_001000221]	-2.31635	4.56E-07
NM_001000814	OLR1220	Rattus norvegicus olfactory receptor 1220 (Olr1220), mRNA [NM_001000814]	-2.32898	4.56E-07

XM_001069077	GPR111	Uncharacterized protein [Source:UniProtKB/TrEMBL;Acc:D4A3T6] [ENSRNOT00000033075]	-2.33393	4.56E-07
NM_001000504	OLR37	Rattus norvegicus olfactory receptor 37 (Olr37), mRNA [NM_001000504]	-2.33745	4.56E-07
NM_001000005	OLR1407	Rattus norvegicus olfactory receptor 1407 (Olr1407), mRNA [NM_001000005]	-2.35884	4.56E-07
NM_001000784	OLR1410	Rattus norvegicus olfactory receptor 1410 (Olr1410), mRNA [NM_001000784]	-2.40083	4.56E-07
NM_001000165	OLR148	Rattus norvegicus olfactory receptor 148 (Olr148), mRNA [NM_001000165]	-2.45057	4.56E-07
NM_001000389	OLR420	Rattus norvegicus olfactory receptor 420 (Olr420), mRNA [NM_001000389]	-2.4783	4.56E-07
NM_001000013	OLR1434	Rattus norvegicus olfactory receptor 1434 (Olr1434), mRNA [NM_001000013]	-2.48564	4.56E-07
NM_001099504	VOM2R79	Rattus norvegicus vomeronasal 2 receptor, 79 (Vom2r79), mRNA [NM_001099504]	-2.48696	4.56E-07
XM_001073458	TCF7	Transcription factor 7, T-cell specific (Predicted), isoform CRA_bUncharacterized protein [Source:UniProtKB/TrEMBL;Acc:D3ZLD0] [ENSRNOT00000008022]	-2.49519	4.56E-07
NM_001030024	SLC19A2	Rattus norvegicus solute carrier family 19 (thiamine transporter), member 2 (Slc19a2), mRNA [NM_001030024]	-2.51142	4.56E-07
NM_001168670	EFNA2	Rattus norvegicus ephrin A2 (Efna2), mRNA [NM_001168670]	-2.52389	4.56E-07
NM_001000812	OLR1236	Rattus norvegicus olfactory receptor 1236 (Olr1236), mRNA [NM_001000812]	-2.55015	4.56E-07
NM_001009650	TAAR5	Rattus norvegicus trace amine-associated receptor 5 (Taar5), mRNA [NM_001009650]	-2.57396	4.56E-07
NM_013169	CD3D	Rattus norvegicus CD3 molecule, delta (Cd3d), mRNA [NM_013169]	-2.57531	4.56E-07
NM_001000923	OLR714	Rattus norvegicus olfactory receptor 714 (Olr714), mRNA [NM_001000923]	-2.58699	4.56E-07
NM_001000114	OLR11	Rattus norvegicus olfactory receptor 11 (Olr11), mRNA [NM_001000114]	-2.58802	4.56E-07
NM_001000986	OLR1132	Rattus norvegicus olfactory receptor 1132 (Olr1132), mRNA [NM_001000986]	-2.59696	4.56E-07
NM_175601	TAAR8B	Rattus norvegicus trace amine-associated receptor 8b (Taar8b), mRNA [NM_175601]	-2.60124	4.56E-07
NM_001000890	OLR1682	Rattus norvegicus olfactory receptor 1682 (Olr1682), mRNA [NM_001000890]	-2.62596	4.56E-07
NM_001000196	OLR211	Rattus norvegicus olfactory receptor 211 (Olr211), mRNA [NM_001000196]	-2.63027	4.56E-07
NM_001000441	OLR1223	Rattus norvegicus olfactory receptor 1223 (Olr1223), mRNA [NM_001000441]	-2.64022	4.56E-07
NM_001077646	CD3G	Rattus norvegicus CD3 molecule, gamma (Cd3g), mRNA [NM_001077646]	-2.64177	4.56E-07
NM_001000635	OLR660	Rattus norvegicus olfactory receptor 660 (Olr660), mRNA [NM_001000635]	-2.66204	4.56E-07
NM_001000518	OLR1283	Rattus norvegicus olfactory receptor 1283 (Olr1283), mRNA [NM_001000518]	-2.6675	4.56E-07
NM_001000882	OLR1122	Rattus norvegicus olfactory receptor 1122 (Olr1122), mRNA [NM_001000882]	-2.6938	4.56E-07
NM_001001007	OLR1664	Rattus norvegicus olfactory receptor 1664 (Olr1664), mRNA [NM_001001007]	-2.69617	4.56E-07
NM_175583	TAAR4	Rattus norvegicus trace amine-associated receptor 4 (Taar4), mRNA [NM_175583]	-2.71292	4.56E-07
NM_001100489	WNT5B	Rattus norvegicus wingless-type MMTV integration site family, member 5B (Wnt5b), mRNA [NM_001100489]	-2.71941	4.56E-07
NM_001000997	OLR689	Rattus norvegicus olfactory receptor 689 (Olr689), mRNA [NM_001000997]	-2.73517	4.56E-07
XM_001064915	OLR1571	PREDICTED: Rattus norvegicus olfactory receptor 1571 (Olr1571), mRNA [XM_001064915]	-2.73754	4.56E-07
NM_012849	GAST	Rattus norvegicus gastrin (Gast), mRNA [NM_012849]	-2.74761	4.56E-07

M31495	SCT	Secretin [Source:UniProtKB/Swiss-Prot;Acc:P11384] [ENSRNOT00000024094]	-2.74975	4.56E-07
NM_198134	BST2	Rattus norvegicus bone marrow stromal cell antigen 2 (Bst2), mRNA [NM_198134]	-2.7574	4.60E-02
NM_001001506	MRGPRD	Rattus norvegicus MAS-related GPR, member D (Mrgprd), mRNA [NM_001001506]	-2.78066	4.56E-07
NM_001000694	OLR16	Rattus norvegicus olfactory receptor 16 (Olr16), mRNA [NM_001000694]	-2.7915	4.56E-07
NM_020087	NOTCH3	Rattus norvegicus notch 3 (Notch3), mRNA [NM_020087]	-2.87925	4.56E-07
NM_001000766	OLR306	Rattus norvegicus olfactory receptor 306 (Olr306), mRNA [NM_001000766]	-2.93705	4.56E-07
NM_001000488	OLR1218	Rattus norvegicus olfactory receptor 1218 (Olr1218), mRNA [NM_001000488]	-3.0052	4.56E-07
NM_001000835	OLR1633	Rattus norvegicus olfactory receptor 1633 (Olr1633), mRNA [NM_001000835]	-3.04681	4.56E-07
NM_001000195	OLR210	Rattus norvegicus olfactory receptor 210 (Olr210), mRNA [NM_001000195]	-3.1134	4.56E-07
NM_001033701	ZEB2	Rattus norvegicus zinc finger E-box binding homeobox 2 (Zeb2), mRNA [NM_001033701]	-3.13125	2.49E-02
NM_001000908	OLR1565	Rattus norvegicus olfactory receptor 1565 (Olr1565), mRNA [NM_001000908]	-3.15916	4.56E-07
NM_022241	PTGDRL	Rattus norvegicus prostaglandin D2 receptor (Ptgdrl), mRNA [NM_022241]	-3.1873	4.56E-07
NM_001108661	CCL19	Rattus norvegicus chemokine (C-C motif) ligand 19 (Ccl19), mRNA [NM_001108661]	-3.21005	4.56E-07
NM_001077644	PTGIR	Rattus norvegicus prostaglandin I2 (prostacyclin) receptor (IP) (Ptgir), mRNA [NM_001077644]	-3.22608	4.56E-07
NM_001000100	OLR1641	Rattus norvegicus olfactory receptor 1641 (Olr1641), mRNA [NM_001000100]	-3.31012	4.56E-07
NM_001000840	OLR1616	Rattus norvegicus olfactory receptor 1616 (Olr1616), mRNA [NM_001000840]	-3.35222	4.56E-07
NM_001000639	OLR650	Rattus norvegicus olfactory receptor 650 (Olr650), mRNA [NM_001000639]	-3.4977	4.56E-07
NM_001001278	OLR382	Rattus norvegicus olfactory receptor 382 (Olr382), mRNA [NM_001001278]	-3.79	4.56E-07
NM_001000129	OLR62	Rattus norvegicus olfactory receptor 62 (Olr62), mRNA [NM_001000129]	-3.82326	4.56E-07
XM_001070788	LOC689435	Uncharacterized protein [Source:UniProtKB/TrEMBL;Acc:D3Z9W7] [ENSRNOT00000044270]	-4.08501	4.56E-07
NM_001000510	OLR1733	Rattus norvegicus olfactory receptor 1733 (Olr1733), mRNA [NM_001000510]	-4.60239	4.56E-07
NM_001000874	OLR1149	Rattus norvegicus olfactory receptor 1149 (Olr1149), mRNA [NM_001000874]	-4.75234	4.56E-07

8. Cognition

NM_017272	ALDH1A7	Rattus norvegicus aldehyde dehydrogenase family 1, subfamily A7 (Aldh1a7), mRNA [NM_017272]	3.930288	8.84E-07
NM_001107569	BBS1	Rattus norvegicus Bardet-Biedl syndrome 1 (Bbs1), mRNA [NM_001107569]	2.105846	8.84E-07
NM_001025061	TAS2R143	Rattus norvegicus taste receptor, type 2, member 143 (Tas2r143), mRNA [NM_001025061]	-2.01041	8.84E-07
NM_001000737	OLR164	Rattus norvegicus olfactory receptor 164 (Olr164), mRNA [NM_001000737]	-2.02686	8.84E-07
NM_134355	POU4F2	Rattus norvegicus POU class 4 homeobox 2 (Pou4f2), mRNA [NM_134355]	-2.06344	8.84E-07
NM_001108198	GUCA1B	Rattus norvegicus guanylate cyclase activator 1B (Guca1b), mRNA [NM_001108198]	-2.07219	8.84E-07
NM_001108118	ITGA5	Rattus norvegicus integrin, alpha 5 (fibronectin receptor, alpha polypeptide) (Itga5), mRNA [NM_001108118]	-2.25615	8.84E-07
NM_012648	SCNN1B	Rattus norvegicus sodium channel, nonvoltage-gated 1, beta (Scnn1b), mRNA [NM_012648]	-2.25721	8.84E-07

NM_031096	GRK1	Rattus norvegicus G protein-coupled receptor kinase 1 (Grk1), mRNA [NM_031096]	-2.35514	8.84E-07
NM_001012219	LHX8	Rattus norvegicus LIM homeobox 8 (Lhx8), mRNA [NM_001012219]	-2.41255	8.84E-07
NM_021669	GHRL	Rattus norvegicus ghrelin/obestatin prepropeptide (Ghrl), mRNA [NM_021669]	-2.60403	4.39E-02
NM_001107619	TMPRSS3	Rattus norvegicus transmembrane protease, serine 3 (Tmprss3), mRNA [NM_001107619]	-2.88409	8.84E-07
NM_001108321	RTP4	Rattus norvegicus receptor (chemosensory) transporter protein 4 (Rtp4), mRNA [NM_001108321]	-4.16531	8.84E-07
XM_219832	AFF2	PREDICTED: Rattus norvegicus AF4/FMR2 family, member 2 (Aff2), mRNA [XM_219832]	-5.1512	8.84E-07
NM_001004099	GJB2	Rattus norvegicus gap junction protein, beta 2 (Gjb2), mRNA [NM_001004099]	-6.22567	8.84E-07
NM_013191	S100B	Rattus norvegicus S100 calcium binding protein B (S100b), mRNA [NM_013191]	-39.6551	8.84E-07
9. Behavioral regulation				
NM_017053	TACR3	Rattus norvegicus tachykinin receptor 3 (Tacr3), mRNA [NM_017053]	4.592168	2.04E-02
NM_012711	ITGAM	Rattus norvegicus integrin, alpha M (Itgam), mRNA [NM_012711]	2.926699	5.13E-03
NM_031688	SNCG	Rattus norvegicus synuclein, gamma (breast cancer-specific protein 1) (Sncg), mRNA [NM_031688]	2.636368	5.33E-03
S53987	CHRNA7	nicotinic receptor alpha 7 subunit [rats, brain, mRNA, 3030 nt]. [S53987]	2.244539	5.33E-03
NM_019282	GREM1	Rattus norvegicus gremlin 1 (Grem1), mRNA [NM_019282]	2.055063	2.04E-02
X15834	CHRNB4	Rattus norvegicus mRNA for muscle like nicotinic acetylcholine receptor subunit (nAChR gene). [X15834]	-2.06121	5.33E-03
NM_012596	LEPR	Rattus norvegicus leptin receptor (Lepr), mRNA [NM_012596]	-2.08406	1.25E-03
NM_013108	ADRB3	Rattus norvegicus adrenoceptor beta 3 (Adrb3), mRNA [NM_013108]	-2.1292	1.25E-03
NM_134336	NLGN3	Rattus norvegicus neuroligin 3 (Nlgn3), mRNA [NM_134336]	-2.16481	1.25E-03
NM_030994	ITGA1	Rattus norvegicus integrin, alpha 1 (Itga1), mRNA [NM_030994]	-2.2508	1.25E-03
NM_012617	OPRD1	Rattus norvegicus opioid receptor, delta 1 (Oprd1), mRNA [NM_012617]	-2.2956	5.33E-03
NM_022797	GRIN2D	Rattus norvegicus glutamate receptor, ionotropic, N-methyl D-aspartate 2D (Grin2d), mRNA [NM_022797]	-2.59165	5.33E-03
10. Transport regulation				
NM_053521	SLC5A7	Rattus norvegicus solute carrier family 5 (choline transporter), member 7 (Slc5a7), mRNA [NM_053521]	2.692472	5.95E-02
NM_031341	SLC7A7	Rattus norvegicus solute carrier family 7 (amino acid transporter light chain, y+L system), member 7 (Slc7a7), mRNA [NM_031341]	2.106627	5.95E-02
BC087709	SLC15A4	Solute carrier family 15 member 4 [Source:UniProtKB/Swiss-Prot;Acc:O09014] [ENSRNOT00000001277]	2.092813	5.95E-02
NM_001025639	LOC292543	Rattus norvegicus similar to solute carrier family 7 (cationic amino acid transporter, y+ system), member 3 (LOC292543), mRNA [NM_001025639]	-2.09633	5.95E-02
FQ211786	SLC6A20	Sodium- and chloride-dependent transporter XTRP3 [Source:UniProtKB/Swiss-Prot;Acc:Q64093] [ENSRNOT00000007467]	-2.20946	5.95E-02
NM_001012227	XK	Rattus norvegicus X-linked Kx blood group (McLeod syndrome) (Xk), mRNA [NM_001012227]	-2.38081	5.95E-02
NM_001106714	SLC7A15	Rattus norvegicus solute carrier family 7 (cationic amino acid transporter, y+ system), member 15 (Slc7a15), mRNA [NM_001106714]	-2.55872	5.95E-02
NM_022866	SLC13A3	Rattus norvegicus solute carrier family 13 (sodium-dependent dicarboxylate transporter), member 3 (Slc13a3), mRNA [NM_022866]	-3.76449	5.95E-02
11. Hormone regulation				
NM_144741	RETN	Rattus norvegicus resistin (Retn), mRNA [NM_144741]	14.69737	2.85E-02
NM_001108037	RDH12	Rattus norvegicus retinol dehydrogenase 12 (all-trans/9-cis/11-cis) (Rdh12), mRNA [NM_001108037]	4.488833	7.76E-02
NM_001135033	TRIM16	Rattus norvegicus tripartite motif-containing 16 (Trim16), mRNA [NM_001135033]	2.547059	2.85E-02

NM_017210	DIO3	Rattus norvegicus deiodinase, iodothyronine, type III (Dio3), mRNA [NM_017210]	2.015739	7.76E-02
NM_130408	CYP26A1	Rattus norvegicus cytochrome P450, family 26, subfamily a, polypeptide 1 (Cyp26a1), mRNA [NM_130408]	-2.00832	7.76E-02
NM_012642	REN	Rattus norvegicus renin (Ren), mRNA [NM_012642]	-2.03807	7.76E-02
NM_172091	GCGR	Rattus norvegicus glucagon receptor (Gcgr), transcript variant 2, mRNA [NM_172091]	-2.05825	2.85E-02
NM_020093	PARK2	Rattus norvegicus parkinson protein 2, E3 ubiquitin protein ligase (Park2), mRNA [NM_020093]	-2.10119	2.85E-02
NM_001198676	CYP2B2	Rattus norvegicus cytochrome P450, family 2, subfamily b, polypeptide 2 (Cyp2b2), mRNA [NM_001198676]	-2.18379	2.85E-02
NM_019630	GIP	Rattus norvegicus gastric inhibitory polypeptide (Gip), mRNA [NM_019630]	-2.20526	2.85E-02
NM_053367	DHH	Rattus norvegicus desert hedgehog (Dhh), mRNA [NM_053367]	-2.22049	2.85E-02
NM_012536	CTRB1	Rattus norvegicus chymotrypsinogen B1 (Ctrb1), mRNA [NM_012536]	-2.24119	2.85E-02
NM_012558	FBP1	Rattus norvegicus fructose-1,6-bisphosphatase 1 (Fbp1), mRNA [NM_012558]	-2.26942	2.85E-02
NM_030988	TG	Rattus norvegicus thyroglobulin (Tg), transcript variant 1, mRNA [NM_030988]	-2.35858	7.76E-02
NM_012909	AQP2	Rattus norvegicus aquaporin 2 (collecting duct) (Aqp2), mRNA [NM_012909]	-2.70795	2.85E-02

12. Development

NM_080407	BMPR2	Rattus norvegicus bone morphogenetic protein receptor, type II (serine/threonine kinase) (Bmpr2), mRNA [NM_080407]	2.439391	5.00E-02
NM_001100889	SHROOM3	Rattus norvegicus shroom family member 3 (Shroom3), mRNA [NM_001100889]	2.08699	2.99E-03
NM_053429	FGFR3	Rattus norvegicus fibroblast growth factor receptor 3 (Fgfr3), mRNA [NM_053429]	-2.0026	2.44E-03
NM_021654	GJA4	Rattus norvegicus gap junction protein, alpha 4 (Gja4), mRNA [NM_021654]	-2.00822	1.86E-04
NM_212545	KRT17	Rattus norvegicus keratin 17 (Krt17), mRNA [NM_212545]	-2.01159	2.44E-03
NM_153627	PROP1	Rattus norvegicus PROP paired-like homeobox 1 (Prop1), mRNA [NM_153627]	-2.04605	2.85E-04
NM_021700	NEUROG3	Rattus norvegicus neurogenin 3 (Neurog3), mRNA [NM_021700]	-2.1207	2.44E-03
NM_001135009	COL4A1	Rattus norvegicus collagen, type IV, alpha 1 (Col4a1), mRNA [NM_001135009]	-2.1646	2.44E-03
NM_001109884	HOXC4	Rattus norvegicus homeo box C4 (Hoxc4), mRNA [NM_001109884]	-2.25763	7.73E-02
NM_053710	PAX3	Rattus norvegicus paired box 3 (Pax3), mRNA [NM_053710]	-2.33376	2.99E-03
NM_012878	SFTPD	Rattus norvegicus surfactant protein D (Sftpd), mRNA [NM_012878]	-2.54395	3.96E-02
NM_001105745	POU2F3	Rattus norvegicus POU class 2 homeobox 3 (Pou2f3), mRNA [NM_001105745]	-2.84536	2.44E-03
NM_001106361	PAX2	Rattus norvegicus paired box 2 (Pax2), mRNA [NM_001106361]	-2.8612	2.99E-03
NM_001108173	TBX18	Rattus norvegicus T-box18 (Tbx18), mRNA [NM_001108173]	-2.9502	2.44E-03
NM_001033701	ZEB2	Rattus norvegicus zinc finger E-box binding homeobox 2 (Zeb2), mRNA [NM_001033701]	-3.13125	2.99E-03
AB118218	MAPK8IP3	Rattus norvegicus JSAP1 mRNA for JNK/SAPK-associated protein 1b, partial cds. [AB118218]	-3.37304	3.96E-02
NM_001105739	PRRX2	Rattus norvegicus paired related homeobox 2 (Prrx2), mRNA [NM_001105739]	-4.04476	2.32E-03
NM_001106942	CDX4	Rattus norvegicus caudal type homeo box 4 (Cdx4), mRNA [NM_001106942]	-4.74028	1.86E-04
NM_001024253	UPK1B	Rattus norvegicus uroplakin 1B (Upk1b), mRNA [NM_001024253]	-4.94311	2.44E-03
