# Supplementary material for: Smad4 SUMOylation is essential for memory formation through upregulation of the skeletal myopathy gene TPM2
Source: BMC Biol. 2017 Nov 28;15:112. doi: 10.1186/s12915-017-0452-9 (PMC5706330; doi:10.1186/s12915-017-0452-9)
Supplement: Supplementary file 6 — Raw data for Fig. 5b. (PDF 42 kb) [file 12915_2017_452_MOESM6_ESM.pdf]

Raw data for Fig.5B (Tpm2 and HPRT RT-qPCR)

Supplementary Table S2

Tpm2											
Group	Flag-vector							Flag-Smad4W			
Repeat no.＼Rat no.	1	2	3	4	5	6	7	1	2	3	4
1	34.51	34.53	34.87	34.68	34.95	34.91	34.57	35.21	35.01	35.06	34.83
2	34.68	34.46	34.98	34.52	34.84	34.83	34.4	35.13	34.72	35.13	34.89
3	35	34.32	34.82	34.34	34.97	34.73	34.42	35.07	34.76	35.11	34.83
Mean	34.73	34.4366667	34.89	34.5133	34.92	34.823333	34.4633	35.1367	34.83	35.1	34.85

HPRT											
Group	Flag-vector							Flag-Smad4W			
Repeat no.＼Rat no.	1	2	3	4	5	6	7	1	2	3	4
1	22.93	23.14	23.09	23.2	23.02	23.08	23.05	23.02	23.06	23.01	23.01
2	23.01	23.1	23.1	23.15	22.99	23.1	23.11	23.05	23.11	23.05	23.05
3	23.13	23.15	23.12	23.01	23.04	23.01	23.05	23.09	23.04	23.06	23.06
Mean	23.02333333	23.13	23.1033333	23.12	23.0167	23.063333	23.07	23.0533	23.07	23.04	23.04

Tpm2/HPRT RT-qPCR summary data

	Traget Ct	eference C	rget Ct-R	Target ΔCt-	fold	fold after normalization
Flag-vector 1	34.73	23.0233	11.7067	0	1	0.92263
Flag-vector 2	34.4366667	23.13	11.3067	-0.4	1.31951	1.21742
Flag-vector 3	34.89	23.1033	11.7867	0.08	0.94606	0.87286
Flag-vector 4	34.5133333	23.12	11.3933	-0.3133333	1.24258	1.14644
Flag-vector 5	34.92	23.0167	11.9033	0.1966667	0.87256	0.80506
Flag-vector 6	34.8233333	23.0633	11.76	0.0533333	0.96371	0.88915

Flag-vector 7	34.4633333	23.07	11.3933	-0.3133333	1.24258	1.14644
Flag-Smad4WT	35.1366667	23.0533	12.0833	0.3766667	0.77022	0.71063
Flag-Smad4WT	34.83	23.07	11.76	0.0533333	0.96371	0.88915
Flag-Smad4WT	35.1	23.04	12.06	0.3533333	0.78277	0.72221
Flag-Smad4WT	34.85	23.04	11.81	0.1033333	0.93088	0.85886
Flag-Smad4WT	35.14	23.0633	12.0767	0.37	0.77378	0.71392
Flag-Smad4WT	34.87	23.1133	11.7567	0.05	0.96594	0.8912
Flag-Smad4WT	34.8366667	23.04	11.7967	0.09	0.93952	0.86683
Flag-Smad4K113RK	36.6066667	23.1667	13.44	1.7333333	0.30076	0.27749
Flag-Smad4K113RK	36.1233333	23.0867	13.0367	1.33	0.39777	0.36699
Flag-Smad4K113RK	36.1433333	23.2167	12.9267	1.22	0.42928	0.39607
Flag-Smad4K113RK	36.8166667	23.0933	13.7233	2.0166667	0.24713	0.22801
Flag-Smad4K113RK	36.93	23.0567	13.8733	2.1666667	0.22272	0.20549
Flag-Smad4K113RK	36.74	23.0533	13.6867	1.98	0.25349	0.23388
Flag-Smad4K113RK	36.02	23.0433	12.9767	1.27	0.41466	0.38258

T			Flag-Smad4K113RK159R						
5	6	7	1	2	3	4	5	6	7
35	34.86	34.81	36.55	36.12	36.18	36.88	36.9	36.72	36
35.27	34.78	34.93	36.5	36.07	36.09	36.84	36.89	36.62	36.03
35.15	34.97	34.77	36.77	36.18	36.16	36.73	37	36.88	36.03
35.14	34.87	34.8367	36.6067	36.1233	36.14333	36.81667	36.93	36.74	36.02

T			Flag-Smad4K113RK159R						
5	6	7	1	2	3	4	5	6	7
23.03	23.19	23.06	23.42	23.05	23.12	23.14	23.17	23.07	23.07
23.09	22.97	23.05	23.13	23.05	23.18	23.08	23	23.02	23.03
23.07	23.18	23.01	22.95	23.16	23.35	23.06	23	23.07	23.03
23.0633	23.1133	23.04	23.1667	23.0867	23.21667	23.09333	23.05667	23.05333	23.04333

