# Supplementary material for: Smad4 SUMOylation is essential for memory formation through upregulation of the skeletal myopathy gene TPM2
Source: BMC Biol. 2017 Nov 28;15:112. doi: 10.1186/s12915-017-0452-9 (PMC5706330; doi:10.1186/s12915-017-0452-9)
Supplement: Supplementary file 7 — Raw data for Fig. 5f. (PDF 41 kb) [file 12915_2017_452_MOESM7_ESM.pdf]

Raw data for Fig. 5F (Tpm2 and HPRT RT-qPCR) Supplementary Table S3

Tpm2

Group	Swim control						Trained 1 day				
Repeat no.＼Rat no.	1	2	3	4	5	6	1	2	3	4	5
1	30.94	31.89	31.86	31.02	31.37	32.05	30.28	30.09	29.96	30.65	30.27
2	31.06	31.49	31.35	31.08	31.38	32.14	30.29	29.94	30.09	31.34	30.56
Mean	31	31.69	31.605	31.05	31.375	32.095	30.285	30.015	30.025	30.995	30.415

HPRT

Group	Swim control						Trained 1 day				
Repeat no.＼Rat no.	1	2	3	4	5	6	1	2	3	4	5
1	23.6114	23.6914	23.5814	23.5814	23.6114	23.6614	23.6214	23.5314	23.5914	23.6914	23.5514
2	23.5914	23.5514	23.5914	23.5314	23.6914	23.5714	23.6914	22.5514	23.35714	23.7014	23.85814
Mean	23.6014	23.6214	23.5864	23.5564	23.6514	23.6164	23.6564	23.0414	23.47427	23.6964	23.70477

Tpm2/HPRT RT-qPCR summary data

	Traget Ct	Reference C	Target Ct-R	Target ΔCt	fold	fold after normalization
Swim control 1	31	23.6014	7.3986	-0.465	1.380317	1.337263
Swim control 2	31.69	23.6214	8.0686	0.205	0.867539	0.840479
Swim control 3	31.605	23.5864	8.0186	0.155	0.898132	0.870118
Swim control 4	31.05	23.5564	7.4936	-0.37	1.292353	1.252042
Swim control 5	31.375	23.6514	7.7236	-0.14	1.101905	1.067535
Swim control 6	32.095	23.6164	8.4786	0.615	0.65293	0.632564
Trained 1 day 1	30.285	23.6564	6.6286	-1.235	2.353813	2.280394
Trained 1 day 2	30.015	23.0414	6.9736	-0.89	1.853176	1.795372
Trained 1 day 3	30.025	23.47427	6.55073	-1.31287	2.484353	2.406861

Trained 1 day 4	30.995	23.6964	7.2986	-0.565	1.479388	1.433243
Trained 1 day 5	30.415	23.70477	6.71023	-1.15337	2.224329	2.154948
Trained 1 day 6	30.88	23.6664	7.2136	-0.65	1.569168	1.520223
Trained 3 day 1	29.77	23.4964	6.2736	-1.59	3.010493	2.916591
Trained 3 day 2	29.3825	23.6814	5.7011	-2.1625	4.4769	4.337257
Trained 3 day 3	29.4075	23.5264	5.8811	-1.9825	3.951773	3.82851
Trained 3 day 4	29.4775	23.5164	5.9611	-1.9025	3.738605	3.621991
Trained 3 day 5	29.555	23.5764	5.9786	-1.885	3.693529	3.578321
Trained 3 day 6	29.855	23.6464	6.2086	-1.655	3.149232	3.051002
Trained 5 day 1	29.13	23.53734	5.59266	-2.27094	4.826375	4.675832
Trained 5 day 2	29.38	23.65234	5.72766	-2.13594	4.395234	4.258139
Trained 5 day 3	29.215	23.66234	5.55266	-2.31094	4.962063	4.807287
Trained 5 day 4	28.865	22.61734	6.24766	-1.61594	3.065112	2.969506
Trained 5 day 5	29.38	23.71734	5.66266	-2.20094	4.597788	4.454375
Trained 5 day 6	29.685	23.62734	6.05766	-1.80594	3.496569	3.387505

	Trained 3 day						Trained 5 day					
6	1	2	3	4	5	6	1	2	3	4	5	6
31.23	29.75	29.33	29.43	29.52	29.57	29.82	29.14	29.42	29.13	28.88	29.44	29.62
30.53	29.79	29.435	29.385	29.435	29.54	29.89	29.12	29.34	29.3	28.85	29.32	29.75
30.88	29.77	29.3825	29.4075	29.4775	29.555	29.855	29.13	29.38	29.215	28.865	29.38	29.685

	Trained 3 day						Trained 5 days					
6	1	2	3	4	5	6	1	2	3	4	5	6
23.6814	23.4614	23.7014	23.5114	23.5314	23.5714	23.6714	23.50234	23.65234	23.63234	22.61234	23.72234	23.60234
23.6514	23.5314	23.6614	23.5414	23.5014	23.5814	23.6214	23.57234	23.65234	23.69234	22.62234	23.71234	23.65234
23.6664	23.4964	23.6814	23.5264	23.5164	23.5764	23.6464	23.53734	23.65234	23.66234	22.61734	23.71734	23.62734

