# Supplementary material for: Smad4 SUMOylation is essential for memory formation through upregulation of the skeletal myopathy gene TPM2
Source: BMC Biol. 2017 Nov 28;15:112. doi: 10.1186/s12915-017-0452-9 (PMC5706330; doi:10.1186/s12915-017-0452-9)
Supplement: Supplementary file 8 — Swim speeds of animals with different treatments. (PDF 48 kb) [file 12915_2017_452_MOESM8_ESM.pdf]

Supplementary Figure 5

A

Swim Speed (cm/sec)	
Cont siRNA	TPM2 siRNA
96.1 ± 3.6	106.2 ± 4.0

B

Swim Speed (cm/sec)		
Lenti-Flag-vector	Lenti-Flag-TPM2WT	Lenti-Flag-TPM2E122K
95.7 ± 4.3	105.1 ± 4.2	102.5 ± 3.5
